# Subsurface fertigation modifies soil–plant–water interactions to improve productivity of cotton–wheat systems under reduced tillage

**DOI:** 10.3389/fpls.2026.1813404

**Published:** 2026-03-26

**Authors:** Kulvir Singh, Yamini Vaddula, Sudhir Kumar Mishra

**Affiliations:** Punjab Agricultural University, Regional Research Station, Faridkot, India

**Keywords:** bulk density, infiltration rate, leaf area index, net photosynthesis, root length density

## Abstract

Excessive reliance on surface flood irrigation accelerates groundwater depletion and degrades soil physical conditions in intensive cropping systems, yet the mechanisms by which irrigation–fertigation strategies influence soil–plant processes under reduced tillage remain poorly understood. We investigated how subsurface drip fertigation (SDF) alters soil physical properties, plant physiological functioning, and system productivity in a low-tilled cotton–wheat rotation over two growing seasons. Subsurface fertigation significantly moderated soil compaction, reducing soil bulk density in the upper (0–15 cm) and subsurface (15–30 cm) layers compared with surface flood irrigation, indicating improved soil structural conditions under localized water and nutrient delivery. Enhanced soil conditions under SDF were associated with improved leaf-level gas exchange, including higher photosynthetic rate, transpiration, and stomatal conductance, which collectively translated into greater crop productivity. Among SDF configurations, lateral placement at 25 cm depth combined with closer emitter spacing (30 cm) optimized root–zone resource availability and physiological performance. Fertigation strategies delivering nitrogen and phosphorus in multiple split applications further strengthened wheat crop responses, highlighting the role of synchronized nutrient–water supply in regulating system productivity. Our findings demonstrate that subsurface fertigation improves soil–plant interactions and resource-use efficiency under reduced tillage, offering a viable pathway to enhance productivity while mitigating pressure on groundwater resources in intensive cropping systems.

## 1 Introduction

Irrigation is essential for agriculture, yet its mismanagement has accelerated resource depletion in India. Agriculture accounts for ~89% of national water use, with groundwater supplying ~62% of irrigation needs (GWRA, 2017). Excessive reliance on groundwater has led to rapid depletion, particularly in Punjab “the Granary of India” which contributes ~26% of wheat and ~5% of cotton production (Brar and Singh, 2022). Cotton and wheat, critical for fiber and food security, face sustainability challenges due to declining water availability, with groundwater levels dropping ~8.91 m over the past decade (Sidhu et al., 2021; Masasi et al., 2020; Grote et al., 2021).Traditional rice–wheat systems dominate >70% of cultivated land, consuming ~200 cm of water annually primarily via surface flood irrigation (Arora and Kukal, 2017). Optimizing water and nutrient use through alternative cropping systems and precise irrigation practices is therefore essential for sustainable intensification (Goyal et al., 2025; Wang et al., 2022). Cotton–wheat cropping systems (CWCS), occupying ~12% of Punjab, are vital for food, fiber, and income security and offer scope for improved resource use efficiency through deficit irrigation and advanced management.

Fertigation, and particularly subsurface drip fertigation (SDF), delivers water and nutrients directly to the root zone, minimizing leaching and volatilization while enhancing yield (Singh et al., 2024). SDF can achieve water use efficiency of 85–90%, reduce evaporation losses, and improve productivity under semi-arid conditions (Tuqan et al., 2020; Gerten et al., 2020; Payero et al., 2005). Despite these benefits, its large-scale adoption in India remains limited, with few studies exploring its effects on soil physical properties, crop physiology, and root dynamics in CWCS (Kaur et al., 2024; Dhayal et al., 2023; Singh and Tomar, 2023).

Despite the demonstrated potential of subsurface drip fertigation (SDF) to improve water and nutrient use efficiency, the mechanisms by which lateral placement depth and emitter configuration regulate soil moisture dynamics, root development, and crop physiological performance under reduced tillage systems remain poorly understood. Most existing studies have focused on yield responses or irrigation scheduling. Limited attention has been given to how the spatial patterns of water and nutrient delivery interact with soil physical properties to shape soil-plant-water processes in intensive cropping systems. In particular, the combined influence of subsurface emitter depth and spacing on soil physical condition and leaf-level physiological functioning has rarely been evaluated in integrated cotton–wheat systems, where contrasting root architectures and seasonal water demands coexist. This knowledge gap restricts the optimization of SDF configurations for sustainable intensification under conditions of declining groundwater availability. We hypothesized that subsurface lateral placement and emitter spacing would differentially modify soil moisture distribution and soil physical properties, thereby regulating root growth and physiological efficiency of cotton and wheat under low-tillage management. To test this hypothesis, a two-year field experiment was conducted to quantify the effects of SDF configurations and fertigation strategies on soil physical attributes, root characteristics, crop physiological responses, and system productivity. The present study aims to: i) Assess the impact of subsurface drip fertigation (SDF) on soil physical properties and physiological traits of cotton and wheat compared to conventional surface flood irrigation (SFM) with soil-applied nutrients. ii) Examine root characteristics under varying lateral depths, emitter spacings, and fertigation levels in contrast to conventional practice. iii) Evaluate the productivity potential of cotton–wheat cropping systems under SDF across different emitter depths, spacings, and nutrient management regimes.

## 2 Materials and methods

### 2.1 Study site

Field experiments were conducted over two years, encompassing four growing seasons i.e., summer 2021 and 2022 for cotton, and winter 2021–22 and 2022–23 for wheat at Punjab Agricultural University, Regional Research Station (RRS), Faridkot (30°40′ N, 74°44′ E; 200 m above MSL). This region experiences hot summer with occasional dust storms and cold winters. Summer temperatures rise from mid-March to June, occasionally exceeding 47 °C, with isolated pre-monsoon showers in May–June. The monsoon, occurring from early July to mid-September, contributes ~70% of annual rainfall but is often interspersed with dry spells. Annual rainfall was 511 mm over 20 days in 2021 and 621.5 mm over 26 days in 2022. Here winters are occasionally influenced by western disturbances, and December–January often experience fog and frost. The experimental soil was sandy loam with slightly alkaline pH. Initial soil physical properties are presented in **Table 1a**.

**Table 1a:** Soil properties of the experimental site.

Soil character	Value	
	0–15 cm	15–30 cm
Soil texture	Loamy	Loamy
pH	8.6	9.2
Electrical conductivity (dS m^-1^)	0.32	0.28
Organic carbon (%)	0.36	0.31
Available N (kg ha^-1^)	263	238
Available P (kg ha^-1^)	28.0	22.1
Available K (kg ha^-1^)	600	564
Bulk density (g m^-3^)	1.53	1.58
Field capacity (%, v/v)	20.0	20.4
Permanent wilting point (%, v/v)	12.1	12.6

**Table 1b:** Fertilizer schedule under different treatments.

Cotton		Wheat	
Treatment	Fertigation schedule (dose ha^-1^)	Treatment	Fertigation schedule (dose ha^-1^)
F_1_- 100% N in 10 similar doses	24.5 kg urea	F_1_- 80% NP in 8 similar doses	19.6 kg urea + 8.2 kg MAP
F_2_- 100% N in 14 similar doses	17.5 kg urea	F_2_- 80% NP in 10 similar doses	15.7 kg urea + 6.6 kg MAP
F_3_- 125% N in 10 similar doses	30.4 kg urea	F_3_-100% NP in 10 similar doses	24.5 kg urea + 10.3 kg MAP
F_4_- 125% N in 14 similar doses	21.7 kg urea	F_4_- 100% NP in 14 similar doses	19.6 kg urea + 8.2 kg MAP
C_2_- 100% N in 10 similar doses	24.5 kg urea	C_2_- 80% NP in 8 similar doses	19.6 kg urea + 8.2 kg MAP
C_1_(SFM)- 100% N (105 kg ha^-1^) in 2 similar doses after 1^st^ and 3^rd^ post-sowing irrigation		C_1_(SFM)- 100% NP (125-62.5 kg ha^-1^) (Full P at sowing, and N in 2 similar doses after 1^st^ and 2^nd^ post-sowing irrigation)	

Where, -100% N, Recommended nitrogen dose (112.5 kg N ha^-1^); 125% N, (140 kg N ha^-1^).

100% NP, Recommended nitrogen and phosphorus dose (125-62.5 kg NP ha^-1^); 80% NP, nitrogen and phosphorus dose (100–50 kg NP ha^-1^);

MAP, Mono ammonium phosphate; SFM, Surface flood method of irrigation along with soil application of fertilizer;

C_1_(SFM), Control 1; C_2_, Control 2; All treatments except Control 1were studied as SDF (Subsurface drip fertigation).

### 2.2 Design of experiment and treatment particulars

**Figure 1:**
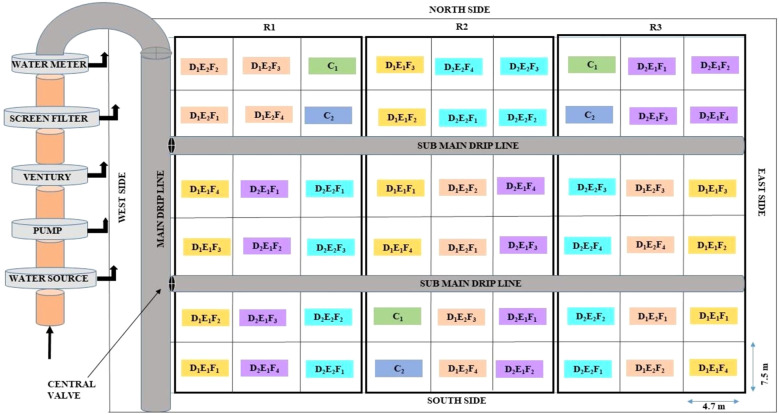
Layout of the experimental field. Treatment details: Main plot (4): Depth of laterals (2) × Emitter spacing (2). Depth of laterals (2): 25 cm (D1) and 30 cm (D2); Emitter to emitter spacing (2): 30 cm (E1) and 40 cm (E2). Sub plot (4): Nitrogen fertigation schedules: F1 - F4. Extra control treatments (2): C_1_ -SFM (Control 1); C_2_ -SSD at 20 cm depth with 20 cm emitter spacing (Control 2).

The field experiment was conducted in a split-plot design with three replications, comprising 18 treatment combinations for each crop. The detailed information has been provided in lay out (**Figure 1**). Bt cotton hybrid (RCH773 BGII) was planted at 67.5 × 75 cm spacing during summer seasons, while wheat cultivar (Unnat PBW343) was sown in 22.5 cm row spacing during winter seasons. Main plot treatments included two subsurface lateral depths: 25 cm (L1) and 30 cm (L2), and two emitter spacings: 30 cm (S1) and 40 cm (S2).

Sub-plot treatments comprised four fertigation schedules as mentioned below:

Cotton: 100% (112.5 kg N ha^-^¹) and 125% recommended N (140 kg N ha^-^¹), applied in ten (F1, F2) or fourteen equal splits (F3, F4).
Wheat: 80% (100–50 kg NP ha^-^¹) and 100% NP (125–62.5 kg NP ha^-^¹), applied in eight (F1, F2) or ten equal splits (F3, F4).

Two control treatments were also studied:

C1 (SFM): Surface flood irrigation with conventional fertilizer application [100% N (105 kg N ha^-^¹)for cotton; 100% NP (125–62.5 kg NP ha^-^¹) for wheat].
C2 (SDF): Subsurface drip fertigation with lateral depth and emitter spacing at 20 cm, and 100% N (112.5 kg N ha^-^¹) for cotton and 80% NP (100–50 kg NP ha^-^¹) for wheat.

All plots, except C1, were sown following reduced preparatory tillage (single round of rotavator) during all crop seasons for cotton. However, no preparatory tillage was given to these plots prior to sowing of wheat crop. C1 plots underwent conventional tillage (two rounds each of cultivator and disc harrow, followed by leveling) and wheat was sown using a conventional drill with full P dose applied with seed. In other plots, wheat was sown using a zero-till drill. Fertilizer schedules and doses are detailed in **Table 1b**.

### 2.3 Drip system installation

After wheat harvest in 2020–21, the experimental field was ploughed twice using a disc harrow and cultivator, followed by leveling. The SDF system was then installed using tractor-mounted laying equipment. PVC lateral pipes were buried at two depths, 25 cm (L1) and 30 cm (L2), with inbuilt emitters spaced at 30 cm (S1) and 40 cm (S2), and lateral spacing of 67.5 cm. Emitters delivered 2.2 L h^-^¹ at 1.5 kPa across all plots. A pre-sowing irrigation of 7.5 cm was applied to fill trenches and ensure uniform leveling. Water meters installed on PVC supply pipes enabled precise quantification of irrigation water, which was recorded for each crop and season (**Supplementary Table 1**). In conventional surface flood plots (C1), irrigation was supplied through separate aboveground PVC laterals. Buffer strips of 2 m width around all experimental plots minimized water distribution variation, while also serving as access pathways for measurements.

### 2.4 Crop care and planting method

During both seasons, cotton has been sown by manual dibbling of 2 seeds hill^-1^, while assuring a uniform depth of 2 inches. After 30 days of sowing (DAS), only one healthy seedling hill^-1^was retained. A planting geometry of 67.5×75 cm was followed, wherein in every plot (4.7m *x* 7.5 m) had total of 5 cotton rows to be served with one water drip line in each row and there were total 11 plants in each row, comprising a total of 55 plants plot^-1^. During *winter*, a PSI of 7.5 cm has been uniformly applied in second fortnight of October, to standing cotton (after first picking) in both the seasons. After final picking (first fortnight of November), cotton plants were cut by sickles and removed. Thereafter, sowing of wheat crop was done with a zero till drill, having rows 22.5 cm wide apart. Each treatment plot had 15 wheat rows, wherein each drip line served irrigation water to 3 rows. Wheat crop has been sown by using a seed rate of 100 kg ha^-1^ in all plots during both years.

### 2.5 Measurements

#### 2.5.1 Soil parameters

##### 2.5.1.1 Initial and final soil fertility status (0–15 cm and 15–30 cm depth)

Composite soil samples were collected after each cotton-wheat crop cycle from all treatment plots at 2 depths, i.e., 0–15 cm & 15–30 cm. These samples were dried in shade, sieved through a 0.2 mm sieve, and used for analyzing soil fertility. To determine available N, the alkaline KMnO_4_ method (Subbiah and Asija, 1956) was employed, while available P was calculated by using the 0.5 M NaHCO_3_ extractable P method (Olsen et al, 1954). Similarly, organic carbon (OC) has been estimated using the rapid titration method (Walkley and Black, 1934).

##### 2.5.1.2 Measurement of soil bulk density and steady state infiltration rate

Soil bulk density has been calculated by employing core method (Black and Hartage, 1986), whereas infiltration rate was measured by using double ring infiltrometer (Bouwer, 1986).

#### 2.5.2 Physiological parameters

##### 2.5.2.1 Net photosynthesis

The photosynthetic rate (Pn) was recorded by employing a portable handheld photosynthesis system Model CI-340 (CID Bio-Science, Inc. Camas, WA, USA), which utilizes an infrared gas analyzer (IRGA).Measurements were recorded on clear sky days between 10:00 and 12:00 noon at 60, 90, 120 and 150 DAS (days after sowing).

##### 2.5.2.2 Transpiration rate

The transpiration rate has been recorded on clear sky days between 10:00 and 12:00 noon at 60, 90, 120 and 150 DAS by using Model CI-340 instrument (CID Bio-Science, Inc. Camas, WA, USA), and the results were expressed in units of mmol m^-^² s^-^¹.

##### 2.5.2.3 Stomatal conductance

The stomatal conductance (G_S_) was measured on clear sky days between 10:00 and 12:00 noon at 60, 90, and 120 DAS using the portable handheld photosynthesis system Model CI-340 (CID Bio-Science, Inc. Camas, WA, USA), and the values for stomatal conductance were expressed in units of mmol m^-^² s^-^¹.

##### 2.5.2.4 Canopy temperature

An infrared radiometer was utilized to record the canopy temperature at 14:30 hours local mean time (LMT) starting from 60 DAS.

##### 2.5.2.5 Chlorophyll content (SPAD)

Five randomly selected plants from each plot of both cotton and wheat experiments were used to measure the chlorophyll content using a Chlorophyll meter (SPAD-502 Plus).

##### 2.5.2.6 Leaf area index

SunScan canopy analyzer by Delta-T Devices Ltd. has been used for recording LAI at regular intervals of 30 days, beginning from 60 DAS on sunny days during noon (11.00 to 1.00 PM).

##### 2.5.2.7 Root length density

Root samples were collected from each plot at two growth stages: cotton at 50% boll formation and near picking, and wheat at booting and harvest. Soil cores were taken at depths of 15, 30, 45, and 60 cm using an auger 3–5 cm from the cotton row and adjacent to drip laterals in wheat. Samples were placed in mesh cloth, gently washed to remove soil, and roots were transferred to petri plates and stored at 4 °C. Root images were scanned using an EPSON flatbed scanner, and root length density (RLD) was analyzed with Biovis root analysis software (Expert Vision Labs Pvt. Ltd., India).

#### 2.5.3 Plant analysis

##### 2.5.3.1 Nitrogen and phosphorus uptake

To measure N uptake, the N content in the above-ground biomass of cotton and the grain and straw of wheat at harvest was determined using Kjeldahl’s apparatus (Piper, 1966). The N uptake was calculated by multiplying the N content with the plant biomass and expressed in kg ha^-1^.To determine P uptake (kg ha^-1^), the phosphorus content in the above-ground biomass of cotton and the grain and straw of wheat was estimated using the Vanado-Molybdo-Phospheric yellow color method in a nitric acid system (Jackson, 1967).

#### 2.5.4 Yield attributes of cotton and wheat

Various yield attributes for cotton were recorded from 5 plants selected at random from every plot while seed cotton yield (SCY) has been recorded from the whole plot. The picking of mature open cotton bolls was carried out manually with two pickings in both years. For each plot, the seed cotton was collected in separate cloth bags and weighed. As per ginning out turn (GOT) values, seed cotton of a particular treatment has been bifurcated into lint yield and seed yield. The ginning out turn was computed using the specified formula.


GOT (%)=[(Weight of lint/weight of seed cotton)×100]

Similarly, for wheat crop, 10 plants from each plot were randomly selected for measurement of yield parameters. After harvesting, the crop produce was kept for sun drying in small bundles in a plot-wise manner. After, threshing grains were collected, and the wheat yield was recorded.

#### 2.5.5 Crop water demand

To estimate the crop water demand, the ETc method has been employed, involving the calculation of water usage by multiplication of the crop coefficient (Kc) with the reference evapotranspiration (ETo). For cotton, Kc value of 0.75 was considered until June, 1.15 for July to August, & 0.70 later on (Singh et al, 2018). Similarly, Kc values for wheat also differed depending on the growth stage, with a value of 0.39 at the beginning stage and 1.26 during the mid to later stages (Kaur et al., 2017). To estimate ETo, procedure adopted by Yamini and Singh (2024) was followed.

#### 2.5.6 Irrigation scheduling

For both crops, irrigation was carried out using the SDF method, with a frequency of every 5 days for cotton and 7 days for wheat. The ETc approach was utilized for determining specific water requirement of each crop, and irrigation water amounting to 80% ETc was applied to both crops, regardless of the treatments. However, for conventional irrigation, SFM was used. The quantity of each irrigation has been accurately measured by fixing water meters on PVC pipes delivering water among all plots. The water usage details over the seasons and crops have been summarized (**Supplementary Table 1**). During second cotton season, only 13 irrigations could be applied as high rainfall (99.6 mm) was recorded on July15^th^ 2022, which forced to skip one irrigation and pending dose was fertigated during third week of August.

### 2.6 Statistical analysis

The data recorded for different parameters have been analyzed by employing single and 3-factor ANOVA (analysis of variance) for a split plot design. For visualizing the differences between treatment means, least significant difference (LSD) test has been used at 5% probability level. The three factor ANOVA using Proc GLM (SAS 9.3, SAS Inc.), has been employed for comparison of the individual and interactive effects between depth of lateral (2), emitter spacing (2), & levels of fertigation (4), whereas both control treatments (C_1_ and C_2_) have been compared with all 16 SDF treatments by using ANOVA for a single factor.

## 3 Results and discussion

### 3.1 Physical parameters of soil

#### 3.1.1 Soil bulk density

**Figure 2:**
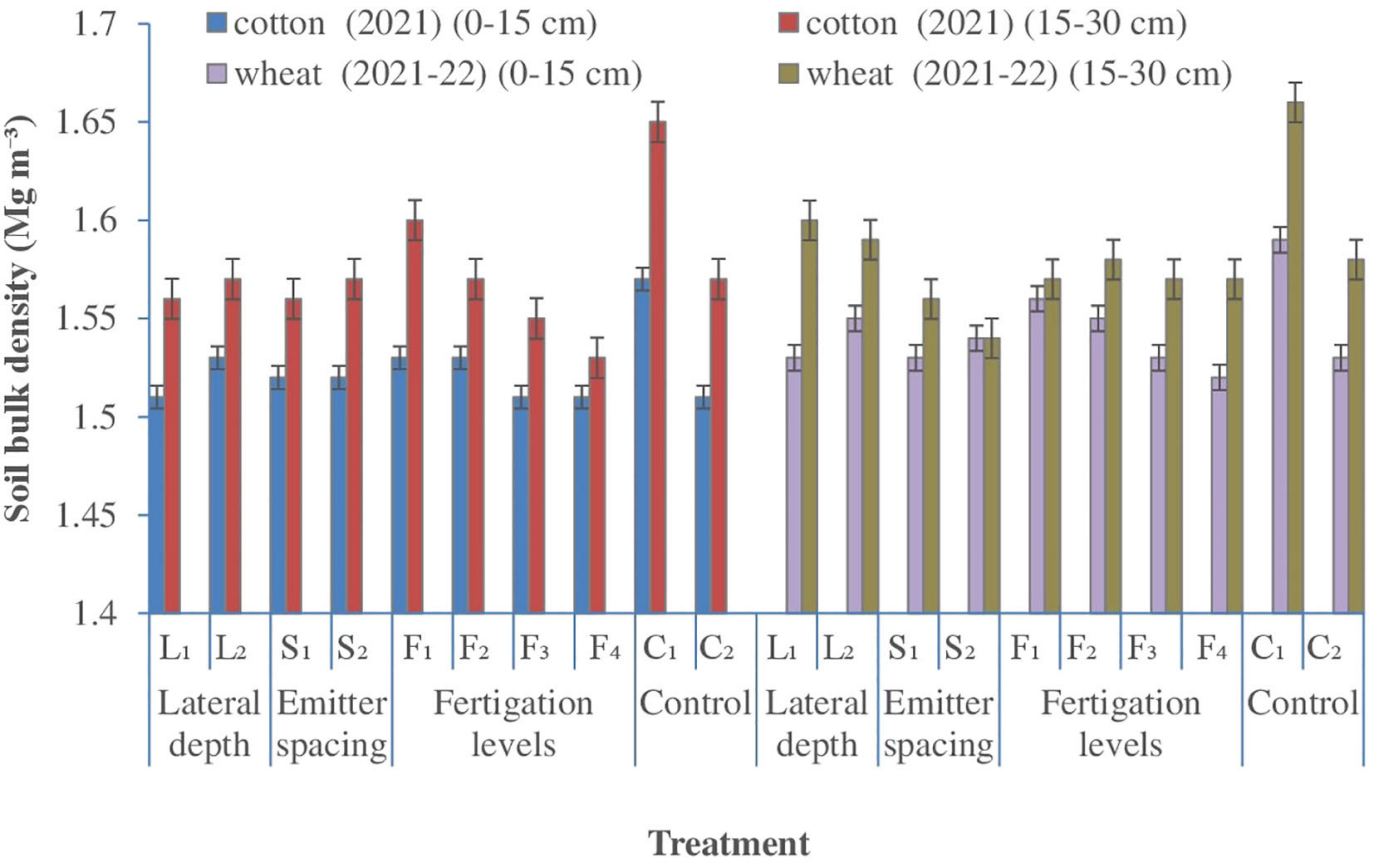
Effect of lateral depth, emitter spacing and fertigation scheduling on soil bulk density. Where: L_1_, Lateral depth of 25 cm; L_2_, Lateral depth of 30 cm; S_1_, Emitter spacing of 30 cm; S_2_, Emitter spacing of 40 cm. For Cotton: F_1_ & F_2_, 100% N (112.5 kg N ha1) fertigation in 10 and 14 similar doses; F_3_ & F_4_, 125% N (140 kg N ha1) fertigation in 10 and 14 similar doses respectively; C_1_, Surface flood with 100% N (105 kg N ha‍1) through manual broadcasting in 2 similar doses (Control 1); C_2_, SDF at 20 cm depth with 100% N (112.5 kg N ha1) in 10 similar doses & emitter spacing at 20 cm (Control 2). For wheat: F_1_ & F_2_, 80% NP (100:50 kg NP ha1) fertigation in 8 and 10 similar doses respectively; F_3_ & F_4_, 100% NP (125:62.5 kg NP ha1) fertigation in 8 and 10 similar doses respectively; C_1_, Surface flood with 100% NP (125:62.5 kg NP ha1) through manual broadcasting in 2 similar doses (Control 1); C_2_, SDF at 20 cm depth with 100% N (100:50 kg NP ha ¨1) in 10 similar doses & emitter spacing at 20 cm (Control 2); SDF, Subsurface drip fertigation.

Soil bulk density responded consistently to subsurface drip fertigation (SDF) configurations across soil depths, with treatment effects becoming more pronounced over successive cropping cycles (**Figure 2**). In general, SDF resulted in lower bulk density compared with surface flood irrigation, indicating improved soil structural condition under localized water and nutrient delivery. The influence of lateral placement depth on bulk density was particularly evident during the second cotton–wheat cycle, suggesting cumulative effects of sustained subsurface wetting and reduced surface disturbance on soil compaction dynamics. Shallower lateral placement (25 cm) reduced bulk density in the surface soil layer (0–15 cm) relative to deeper placement (30 cm), reflecting more favorable moisture distribution and reduced mechanical impedance near the active root zone. Across seasons, bulk density increased gradually from cotton to wheat, with larger increases observed following the wheat crop, likely due to greater soil settling and traffic during the winter season responses previously reported for cotton-based cropping systems (Singh et al., 2019; Omololu et al., 2020). In contrast, emitter spacing exerted only a marginal influence on bulk density, indicating that lateral depth and fertigation strategy were the primary drivers of changes in soil physical condition.

Fertigation effects were more evident in the subsurface layer (15–30 cm), where increased nutrient supply was associated with reduced bulk density. This response can be attributed to enhanced root proliferation and greater belowground biomass production under higher nitrogen and phosphorus availability, which promote aggregation and porosity through root turnover and organic matter inputs (Hati et al., 2007). In addition, increased nutrient availability and higher Root length density may stimulate microbial and soil faunal activity via greater soil organic carbon inputs, further improving pore continuity and reducing bulk density (Haynes and Naidu, 1998). Similar reductions in soil bulk density under higher fertilization regimes have been reported in intensive cereal-based cropping systems (Banashree et al., 2015). Across both soil depths, surface flood irrigation consistently recorded the highest bulk density, confirming the adverse effects of conventional irrigation on soil physical properties. After two cropping cycles, bulk density under surface flood irrigation exceeded that under subsurface drip fertigation by approximately 4–5%, consistent with earlier observations of compaction under flood-irrigated systems (Singh et al., 2023). Collectively, these results demonstrate that subsurface fertigation particularly with shallow lateral placement and optimized nutrient supply can progressively improve soil structural conditions, thereby creating a more favorable root-zone environment under reduced tillage management.

#### 3.1.2 Steady state infiltration rate

**Figure 3:**
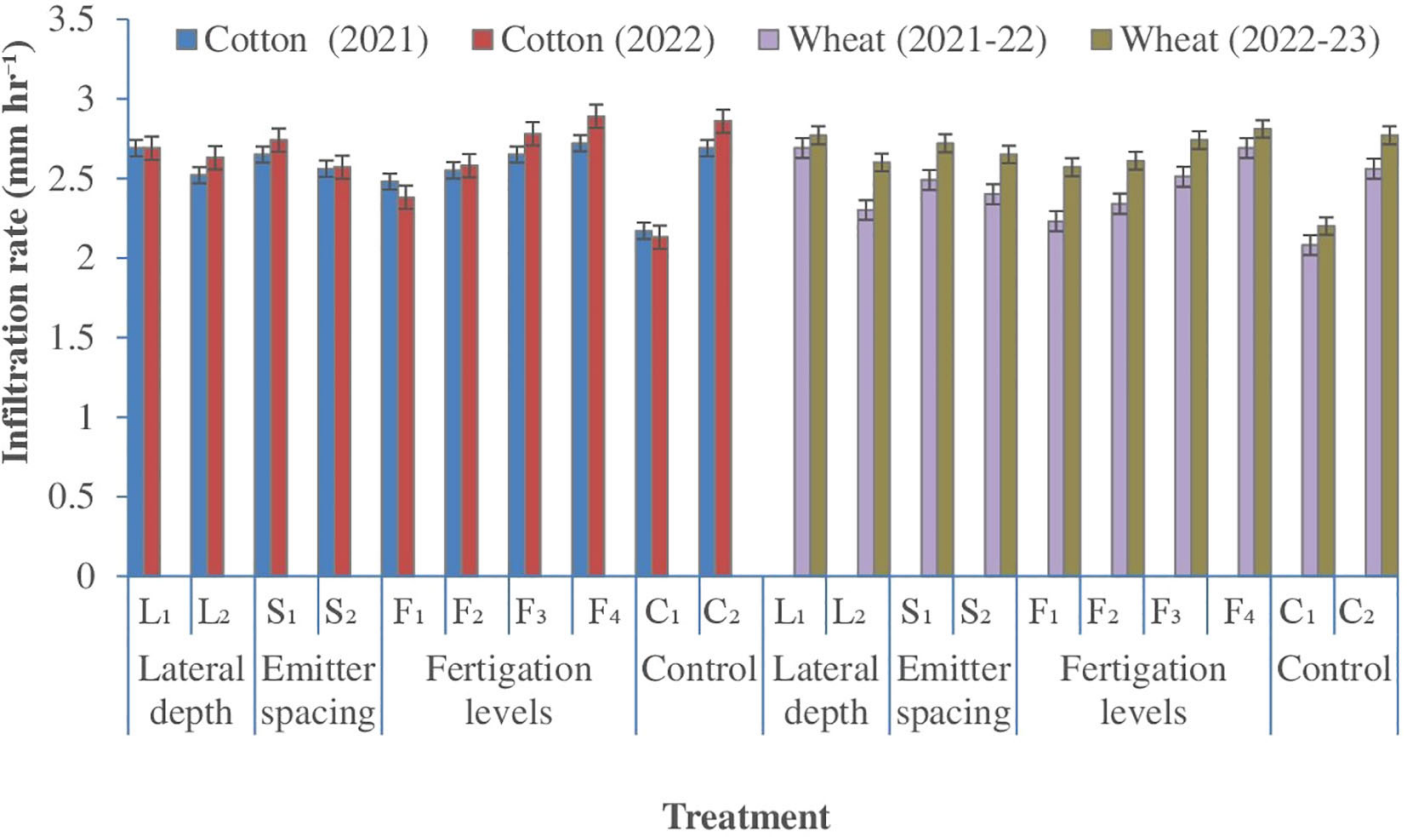
Effect of lateral depth, emitter spacing and fertigation scheduling on steady state infiltration rate. Where: L_1_, Lateral depth of 25 cm; L_2_, Lateral depth of 30 cm; S_1_, Emitter spacing of 30 cm; S_2_, Emitter spacing of 40 cm. For Cotton: F_1_ & F_2_, 100% N (112.5 kg N ha1) fertigation in 10 and 14 similar doses; F_3_ & F_4_, 125% N (140 kg N ha1) fertigation in 10 and 14 similar doses respectively; C_1_, Surface flood with 100% N (105 kg N ha‍1) through manual broadcasting in 2 similar doses (Control 1); C_2_, SDF at 20 cm depth with 100% N (112.5 kg N ha1) in 10 similar doses & emitter spacing at 20 cm (Control 2). For wheat: F_1_ & F_2_, 80% NP (100:50 kg NP ha1) fertigation in 8 and 10 similar doses respectively; F_3_ & F_4_, 100% NP (125:62.5 kg NP ha1) fertigation in 8 and 10 similar doses respectively; C_1_, Surface flood with 100% NP (125:62.5 kg NP ha1) through manual broadcasting in 2 similar doses (Control 1); C_2_, SDF at 20 cm depth with 100% N (100:50 kg NP ha ¨1) in 10 similar doses & emitter spacing at 20 cm (Control 2); SDF, Subsurface drip fertigation.

**Figure 4:**
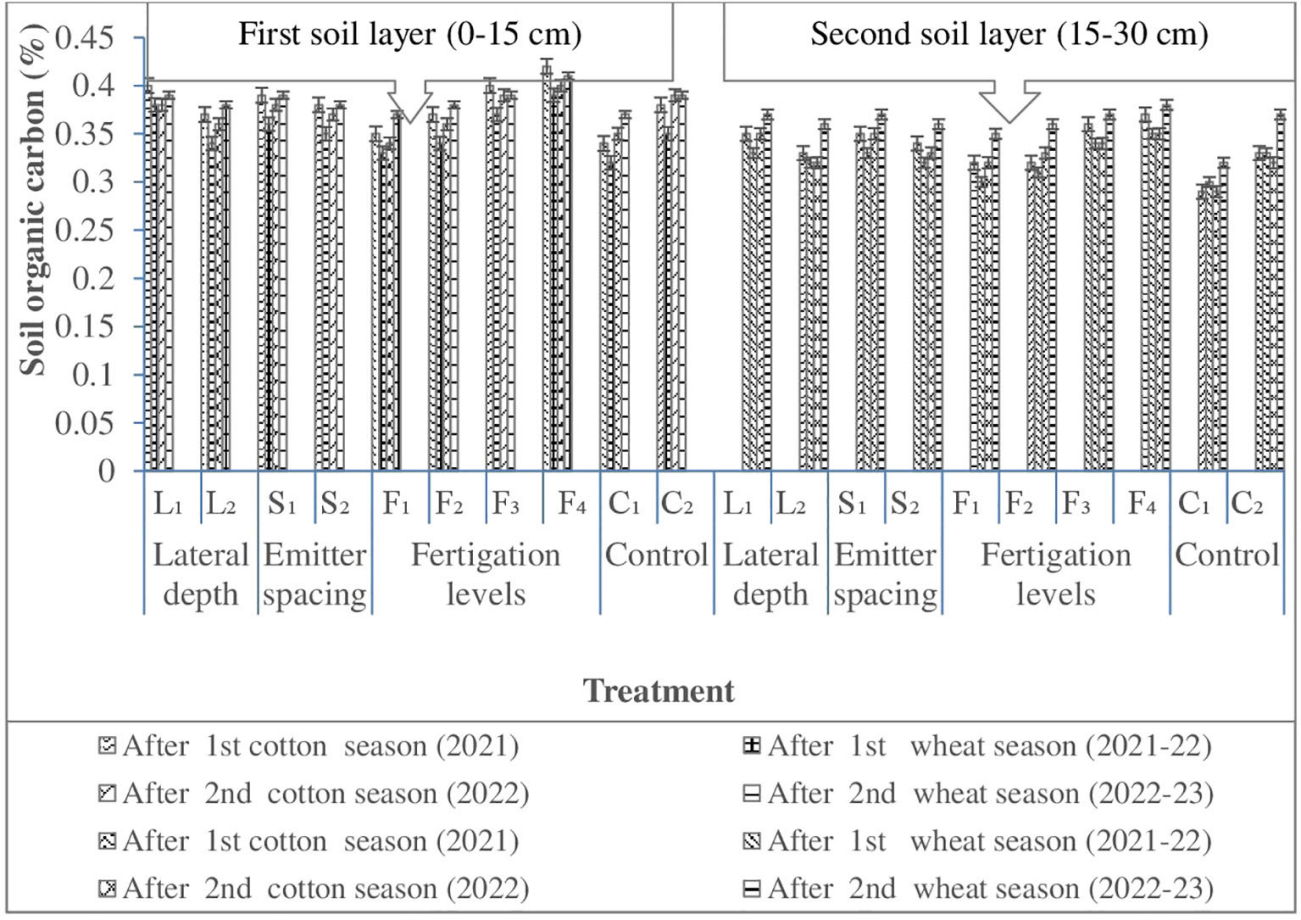
Effect of lateral depth, emitter spacing and fertigation scheduling on soil organic carbon. Where: L_1_, Lateral depth of 25 cm; L_2_, Lateral depth of 30 cm; S_1_, Emitter spacing of 30 cm; S_2_, Emitter spacing of 40 cm. For Cotton: F_1_ & F_2_, 100% N (112.5 kg N ha1) fertigation in 10 and 14 similar doses; F_3_ & F_4_: 125% N (140 kg N ha1) fertigation in 10 and 14 similar doses respectively; C_1_: Surface flood with 100% N (105 kg N ha‍1) through manual broadcasting in 2 similar doses (Control 1); C_2_: SDF at 20 cm depth with 100% N (112.5 kg N ha1) in 10 similar doses & emitter spacing at 20 cm (Control 2). For wheat: F_1_ & F_2_, 80% NP (100:50 kg NP ha1) fertigation in 8 and 10 similar doses respectively; F_3_ & F_4_, 100% NP (125:62.5 kg NP ha1) fertigation in 8 and 10 similar doses respectively; C_1_, Surface flood with 100% NP (125:62.5 kg NP ha1) through manual broadcasting in 2 similar doses (Control 1); C_2_, SDF at 20 cm depth with 100% N (100:50 kg NP ha ¨1) in 10 similar doses & emitter spacing at 20 cm (Control 2); SDF, Subsurface drip fertigation.

Steady-state infiltration rate was strongly influenced by SDF configuration and nutrient supply, whereas emitter spacing exerted no significant effect (**Figure 3**). Shallow lateral placement (25 cm) consistently enhanced SSIR compared with deeper placement (30 cm) across cropping seasons, with the largest relative increase observed following the wheat crop. This response indicated that subsurface wetting closer to the active root zone progressively improves soil hydraulic functioning under reduced tillage. Across both cotton and wheat, SSIR increased with higher fertigation levels, reflecting the cumulative effects of synchronized water and nutrient delivery on soil physical condition. The highest fertigation treatment consistently recorded superior SSIR, which coincided with greater soil organic carbon accumulation and reduced soil bulk density (**Figure 4**). These coupled responses suggest that improved aggregation and pore continuity under enhanced nutrient availability facilitated greater water transmission through the soil profile. Increased organic inputs and root turnover under higher nitrogen and phosphorus supply likely stimulated soil biotic activity, possibly contributing to the development of stable macro-porosity and improved infiltration capacity (Bhattacharyya et al., 2007; Brar et al., 2015).Irrespective of SDF configuration, SSIR was markedly higher under subsurface drip irrigation than values under surface flood. Surface flood irrigation consistently exhibited the lowest infiltration rates, reflecting the adverse effects of prolonged surface wetting, soil sealing, and repeated tillage on soil structure. Similar reductions in infiltration under flood-irrigated systems have been attributed to compaction and pore collapse in intensively managed cropping systems (Singh et al., 2023). Collectively, these results indicate that subsurface fertigation enhances soil hydraulic properties by improving aggregation and pore connectivity, thereby promoting more efficient water entry into the soil profile under intensive cotton–wheat rotations.

#### 3.1.3 Soil organic carbon

Soil organic carbon is a key indicator of soil quality and long-term agricultural sustainability, as it governs aggregate stability, water retention, and nutrient cycling (Liu et al., 2006). Across the CWCS, SOC dynamics were primarily influenced by subsurface lateral placement depth and fertigation strategy, whereas emitter spacing exerted only a marginal effect (**Figure 4**). Shallow lateral placement (25 cm) consistently resulted in higher SOC concentrations than deeper placement (30 cm) in soil layers, indicating greater carbon inputs and retention within the active root zone under near-surface subsurface wetting. SOC increased progressively over cropping seasons across all treatments, with more pronounced accumulation observed under higher fertigation levels. This trend reflects enhanced crop growth and greater belowground carbon inputs under synchronized water and nutrient supply. The highest fertigation treatment consistently recorded the greatest SOC accumulation in both surface and subsurface soil layers, suggesting that increased nutrient availability stimulated biomass production, root turnover, and residue inputs, thereby strengthening soil carbon pools. Improved nitrogen availability under fertigation may also have accelerated microbial activity and organic matter transformation, further contributing to SOC stabilization. Therefore, a cumulative beneficiary effect was evident under SDF.

In contrast, surface flood irrigation recorded the lowest SOC levels by the end of the cropping cycle, reflecting lower nutrient-use efficiency and reduced biomass inputs under conventional water management. The comparatively subdued SOC accumulation under flood irrigation underscores the importance of localized fertigation in enhancing carbon inputs and retention in intensively managed cropping systems. Collectively, these results demonstrate that subsurface drip fertigation promotes SOC sequestration by improving nutrient synchronization and root-zone conditions, thereby reinforcing the link between soil carbon dynamics and improved soil physical and hydraulic properties under reduced tillage cotton–wheat systems.

### 3.2 Physiological parameters of cotton

#### 3.2.1 Net photosynthesis

**Figure 5:**
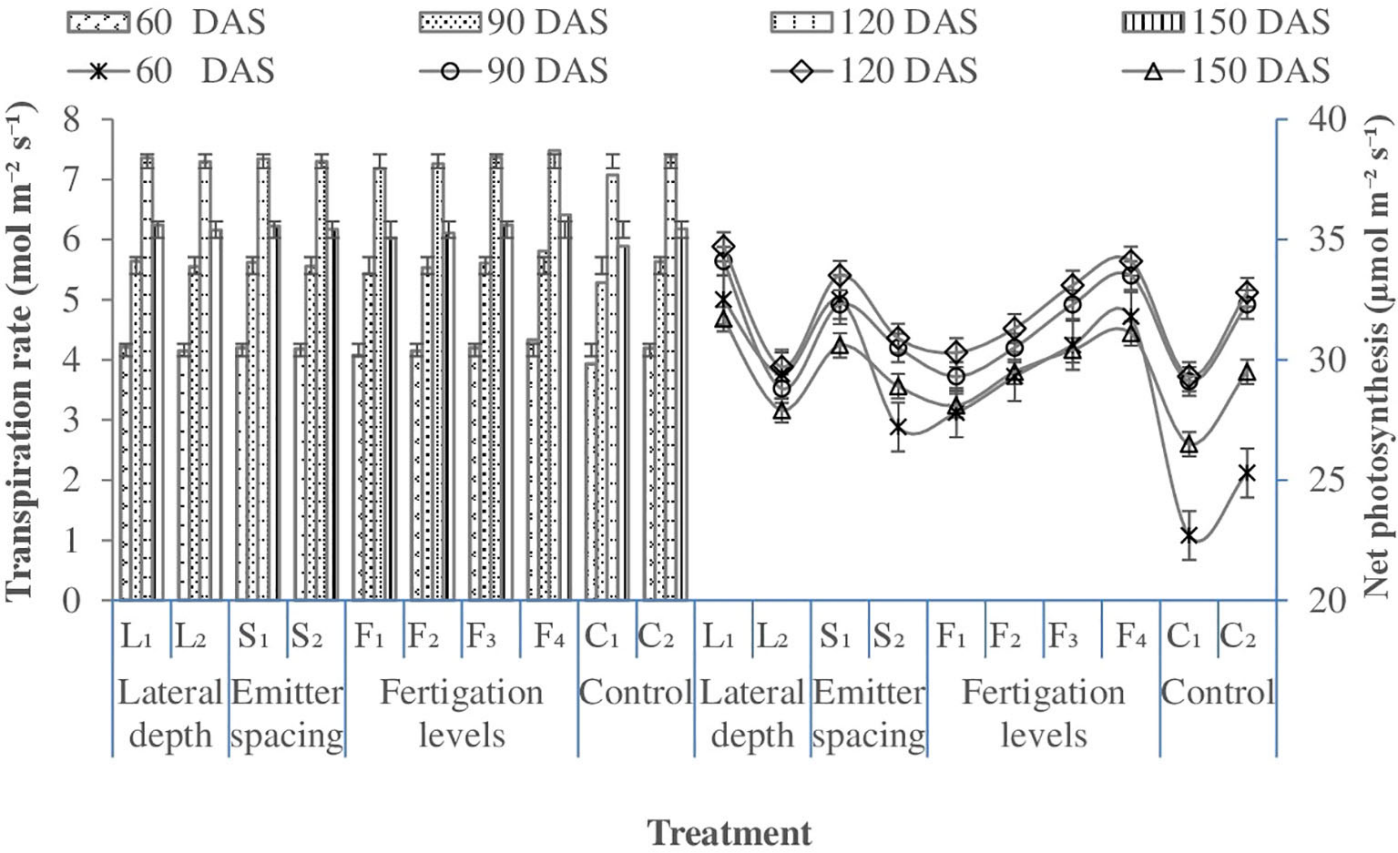
Effect of lateral depth, emitter spacing and fertigation scheduling on net photosynthesis and transpiration rate of cotton (Pooled mean of 2 years). Where: L_1_, Lateral depth of 25 cm; L_2_, Lateral depth of 30 cm; S_1_, Emitter spacing of 30 cm; S_2_, Emitter spacing of 40 cm, F_1_ & F_2_, 100% N (112.5 kg N ha1) fertigation in 10 and 14 similar doses; F_3_ & F_4_: 125% N (140 kg N ha1) fertigation in 10 and 14 similar doses respectively; C_1_: Surface flood with 100% N (105 kg N ha‍1) through manual broadcasting in 2 similar doses (Control 1); C_2_: SDF at 20 cm depth with 100% N (112.5 kg N ha1) in 10 similar doses & emitter spacing at 20 cm (Control 2). For wheat: F_1_ & F_2_, 80% NP (100:50 kg NP ha1) fertigation in 8 and 10 similar doses respectively; F_3_ & F_4_, 100% NP (125:62.5 kg NP ha1) fertigation in 8 and 10 similar doses respectively; C_1_, Surface flood with 100% NP (125:62.5 kg NP ha1) through manual broadcasting in 2 similar doses (Control 1); C_2_, SDF at 20 cm depth with 100% N (100:50 kg NP ha ¨1) in 10 similar doses & emitter spacing at 20 cm (Control 2); SDF, Subsurface drip fertigation; DAS, days after sowing.

Net photosynthetic rate was strongly influenced by SDF configuration and nitrogen supply across growth stages (**Figure 5**). Shallow lateral placement (25 cm) consistently enhanced photosynthesis compared with deeper placement (30 cm), particularly during the mid to late growth stages, indicating improved root-zone water and nutrient availability under near-surface subsurface wetting. Enhanced photosynthetic performance under shallow lateral placement reflects more effective coupling between soil moisture availability and nutrient uptake, which sustains leaf metabolic activity and carbon assimilation.

Emitter spacing exerted a comparatively smaller influence on photosynthesis. Closer emitter spacing (30 cm) tended to promote higher photosynthetic rates during early growth stages, although this effect diminished later in the season and was not consistently significant. This pattern suggests that uniform early-season moisture distribution may transiently enhance physiological performance, while sustained photosynthetic activity later in the season is primarily governed by lateral placement depth and nutrient supply rather than emitter density (Chen et al., 2018b). Nitrogen fertigation exerted a pronounced and consistent effect on photosynthesis across both seasons, with higher nutrient supply resulting in progressively greater photosynthetic rates. Split application of nitrogen through fertigation maintained higher photosynthetic activity throughout the crop cycle, highlighting the importance of its synchronized availability with crop demand. Elevated photosynthesis under higher nitrogen levels can be attributed to increased chlorophyll content, and improved stomatal conductance, all of which are closely linked to leaf nitrogen status (Wullschleger and Oosterhuis, 1990; Pettigrew and Gerik, 2007).Among irrigation treatments, surface flood irrigation consistently exhibited lower photosynthetic rates than subsurface drip fertigation. Reduced photosynthesis under flood irrigation likely reflects suboptimal nitrogen availability and transient water stress conditions, which accelerate leaf senescence and limit assimilate supply to developing sinks (Zhao and Oosterhuis, 2000). Similar reductions in photosynthetic activity under water-limited or poorly synchronized moisture conditions have been reported in cotton, emphasizing the sensitivity of canopy carbon assimilation to root-zone water status (Singh et al., 2022b).

Collectively, these results demonstrate that enhanced photosynthesis under subsurface fertigation arises from the combined effects of improved root-zone moisture distribution and sustained nitrogen availability, reinforcing the role of SDF in strengthening physiological efficiency and carbon assimilation in cotton under reduced tillage systems.

#### 3.2.2 Transpiration rate

Transpiration rate was significantly influenced by fertigation schedules, whereas lateral placement depth and emitter spacing did not exert a measurable effect (**Figure 5**). Progressive enhancement in nutrient supply from F1 to F4 resulted in a consistent increase in transpiration across all growth stages, indicating a strong linkage between nitrogen availability and canopy gas exchange processes. Higher transpiration under elevated fertigation levels can be attributed to improved leaf nitrogen status, which promoted chlorophyll synthesis and increased leaf area index (**Table 2**). Enhanced canopy development under higher nitrogen supply facilitated greater radiation interception and carbon assimilation, thereby intensifying stomatal activity and transpirational water loss (Silvertooth et al., 2011). The observed coupling between increased photosynthetic rate and transpiration suggests that nitrogen-induced improvements in canopy structure and function strengthened plant–atmosphere water vapor exchange, as reported by Manzoor et al. (2021).

**Table 2:** Effect of lateral depth, emitter spacing and fertigation scheduling on chlorophyll content and leaf area index of cotton (pooled mean of 2 years).

Lateral depth (L)	Emitter spacing (S)	Fertigation levels (F)	Chlorophyll content (SPAD value)				Leaf area index			
			60 DAS	90 DAS	120 DAS	150 DAS	60 DAS	90 DAS	120 DAS	150 DAS
L_1_ (25 cm)	S_1_ (30 cm)	F_1_	36.3	39.1	44.0	39.0	2.03	2.90	3.79	3.27
		F_2_	38.6	41.6	45.4	41.8	2.19	2.97	3.95	3.49
		F_3_	41.0	43.1	47.2	44.2	2.45	3.15	4.25	3.65
		F_4_	42.2	43.9	48.2	45.9	2.65	3.39	4.59	4.00
	S_2_ (40 cm)	F_1_	35.8	38.8	43.0	37.8	1.92	2.79	3.67	3.21
		F_2_	37.6	41.0	43.7	40.7	2.13	2.91	3.78	3.30
		F_3_	39.7	42.1	46.2	42.3	2.35	3.02	3.94	3.52
		F_4_	40.6	43.1	47.6	44.6	2.46	3.21	4.22	3.69
L_2_ (30 cm)	S_1_ (30 cm)	F_1_	35.6	38.3	41.6	36.7	1.77	2.73	3.64	2.98
		F_2_	36.6	40.6	43	39.3	2.06	2.90	3.80	3.16
		F_3_	38.2	40.7	44.5	40.7	2.20	2.99	3.89	3.41
		F_4_	38.3	41.6	45.1	42.9	2.36	3.16	4.14	3.53
	S_2_ (40 cm)	F_1_	34.6	36.6	40.8	36.1	1.68	2.38	3.34	2.90
		F_2_	35.4	38.8	42.5	38.3	1.96	2.78	3.59	3.06
		F_3_	36.8	40.0	44.3	39.9	2.08	2.89	3.69	3.30
		F_4_	37.5	41.0	45.0	41.9	2.21	3.00	3.97	3.37
L_1_			39.0	41.6	45.7	42	2.27	3.04	4.03	3.52
L_2_			36.6	39.7	43.1	39.5	2.04	2.85	3.76	3.21
S_1_			38.3	41.1	44.7	41.3	2.22	3.02	4.01	3.44
S_2_			37.2	40.2	44.1	40.2	2.12	2.88	3.79	3.31
F_1_			35.6	38.2	42.0	37.4	1.85	2.70	3.61	3.09
F_2_			37.0	40.5	43.6	40.0	2.09	2.89	3.78	3.25
F_3_			38.9	41.5	45.5	41.8	2.27	3.01	3.95	3.47
F_4_			39.7	42.4	46.5	43.8	2.42	3.19	4.23	3.65
C_1_			33.3	35.8	40.6	38.6	1.74	2.30	3.26	2.75
C_2_			39.4	41.6	44.7	42.7	2.14	2.92	3.85	3.36
CD (p=0.05)										
Lateral depth (L)			1.3	1.8	1.3	2.2	0.07	0.08	0.13	0.11
Emitter spacing (S)			1.3	NS	NS	NS	0.07	0.08	0.13	0.11
Fertigation levels (F)			1.3	1.6	1.6	2.0	0.08	0.10	0.12	0.11
Interactions			NS	NS	NS	NS	NS	NS	NS	NS
C_1_ vs C_2_			1.9	3.0	3.7	3.8	0.11	0.14	0.25	0.17
Treatments vs Controls			1.9	3.0	NS	NS	0.11	0.14	0.26	0.17

Where:

F_1_& F_2_: 100% N (112.5 kg N ha^-1^) fertigation in 10 and 14 similar doses;

F_3_& F_4_: 125% N (140 kg N ha^-1^) fertigation in 10 and 14 similar doses respectively;

C_1_(SFM):Surface flood with 100% N (105 kg N ha^-1^) through manual broadcasting in 2 similar doses (Control 1);.

C_2_: SDF at 20 cm depth with 100% N (112.5 kg N ha^-1^) in 10 similar doses& emitter spacing at 20 cm (Control 2);

SDF: Subsurface drip fertigation;

DAS: days after sowing.

Among irrigation treatments, surface flood method consistently exhibited the lowest transpiration rates. Reduced transpiration under this treatment likely resulted from intermittent water stress and restricted canopy development relative to subsurface drip fertigation. Under such conditions, stomatal closure acts as a protective mechanism to limit water loss, consequently reducing transpiration. Comparable responses have been documented in cotton, where gas exchange parameters under rainfall-dependent or poorly regulated moisture regimes resembled those of water-limited systems rather than optimally irrigated crops (Chastain et al., 2014).Overall, these findings demonstrate that enhanced transpiration under subsurface drip fertigation is primarily driven by improved nitrogen availability and canopy development, reinforcing the central role of fertigation scheduling in regulating cotton physiological performance under CWCS.

#### 3.2.3 Stomatal conductance

Stomatal conductance was higher under a shallower lateral placement (L1:25 cm) and closer emitter spacing (30 cm) compared to deeper placement and wider spacing (**Table 3**). The shallow lateral depth (L1) consistently recorded the highest stomatal conductance across growth stages, indicating improved soil moisture availability and favorable root-zone conditions. Similar responses under closer emitter spacing reflect more uniform moisture and nutrient distribution within the active root zone, thereby facilitating enhanced gas exchange. These findings are in agreement with Chen et al. (2018b), who reported improved stomatal conductance and gas exchange parameters under shallower subsurface drip irrigation depths. Fertigation levels exerted a significant influence on stomatal conductance, with values increasing progressively from 60 to 120 DAS and declining thereafter. Higher nitrogen supply (125% N), particularly when applied in 14 equal splits (F4), resulted in maximum stomatal conductance at peak vegetative growth (120 DAS) during both years. Enhanced stomatal conductance under higher N application may be attributed to improved nutrient availability, leading to increased chlorophyll content, greater photosynthetic activity, and better canopy development.

**Table 3:** Effect of lateral depth, emitter spacing and fertigation scheduling on stomatal conductance and canopy temperature of cotton (pooled mean of 2 years).

Lateral depth (L)	Emitter spacing (S)	Fertigation levels (F)	Stomatal conductance (mol m^-2^ s^-1^)				Canopy temperature (°C)			
			60 DAS	90 DAS	120 DAS	150 DAS	60 DAS	90 DAS	120 DAS	150 DAS
L_1_ (25 cm)	S_1_ (30 cm)	F_1_	0.51	0.52	0.53	0.50	34.9	32.6	31.8	29.9
		F_2_	0.52	0.54	0.55	0.52	34.5	32.1	31.6	29.1
		F_3_	0.54	0.55	0.57	0.54	33.2	31.3	31.2	28.2
		F_4_	0.54	0.56	0.58	0.55	32.9	30.0	29.3	27.7
	S_2_ (40 cm)	F_1_	0.45	0.47	0.49	0.45	34.8	33.0	32.1	31.4
		F_2_	0.48	0.51	0.52	0.49	34.3	32.7	31.1	29.8
		F_3_	0.50	0.53	0.55	0.52	33.1	31.8	30.7	29.0
		F_4_	0.53	0.55	0.55	0.52	32.9	31.1	29.7	28.5
L_2_ (30 cm)	S_1_ (30 cm)	F_1_	0.39	0.42	0.44	0.42	34.1	32.7	31.4	29.7
		F_2_	0.42	0.45	0.47	0.44	33.7	32.2	30.7	28.9
		F_3_	0.43	0.48	0.50	0.47	33.1	31.1	29.9	28.6
		F_4_	0.46	0.49	0.51	0.49	32.6	30.4	29.6	28.1
	S_2_ (40 cm)	F_1_	0.36	0.39	0.42	0.40	35.0	33.1	32.8	30.5
		F_2_	0.39	0.41	0.44	0.40	34.5	32.4	31.2	29.5
		F_3_	0.41	0.44	0.46	0.44	33.8	31.1	30.6	28.8
		F_4_	0.43	0.47	0.49	0.45	33.3	31.0	30.0	28.4
L_1_			0.51	0.53	0.54	0.51	33.8	31.8	30.9	29.2
L_2_			0.41	0.44	0.46	0.44	33.8	31.7	30.8	29.1
S_1_			0.48	0.50	0.52	0.49	33.6	31.6	30.7	28.8
S_2_			0.44	0.47	0.49	0.46	34.0	32.0	31.0	29.2
F_1_			0.43	0.45	0.47	0.44	34.7	32.9	32.0	30.4
F_2_			0.45	0.48	0.49	0.46	34.2	32.3	31.1	29.3
F_3_			0.47	0.50	0.52	0.49	33.3	31.3	30.6	28.6
F_4_			0.49	0.52	0.53	0.50	32.9	30.6	29.7	28.2
C_1_			0.32	0.35	0.38	0.35	36.0	34.1	32.9	32.2
C_2_			0.50	0.53	0.52	0.50	34.1	31.9	30.2	28.5
LSD (p=0.05)										
Lateral depth (L)			0.01	0.02	0.01	0.01	NS	NS	NS	NS
Emitter spacing (S)			0.01	0.02	0.01	0.01	NS	NS	NS	0.4
Fertigation levels (F)			0.02	0.02	0.02	0.01	0.7	0.8	0.8	0.5
Interactions			NS	NS	NS	NS	NS	NS	NS	NS
C_1_ vs C_2_			0.01	0.01	0.03	0.01	1.1	1.2	0.4	1.0
Treatments vs Controls			0.01	0.01	0.03	0.01	1.1	1.2	0.4	1.0

Where:

F_1_& F_2_, 100% N (112.5 kg N ha^-1^) fertigation in 10 and 14 similar doses;

F_3_& F_4_, 125% N (140 kg N ha^-1^) fertigation in 10 and 14 similar doses respectively;

C_1_(SFM), Surface flood with 100% N (105 kg N ha^-1^) through manual broadcasting in 2 similar doses (Control 1);

C_2_, SDF at 20 cm depth with 100% N (112.5 kg N ha^-1^) in 10 similar doses& emitter spacing at 20 cm (Control 2);

SDF, Subsurface drip fertigation;

DAS, days after sowing.

All SDF treatments exhibited significantly higher stomatal conductance than surface flood irrigation (C1). The markedly lower values observed under C1 indicate moisture stress and heterogeneous soil water distribution within the root zone, resulting in partial stomatal closure. These differences highlight the critical role of subsurface drip fertigation in maintaining favorable soil–plant–atmosphere continuum conditions.

#### 3.2.4 Canopy temperature

**Figure 6:**
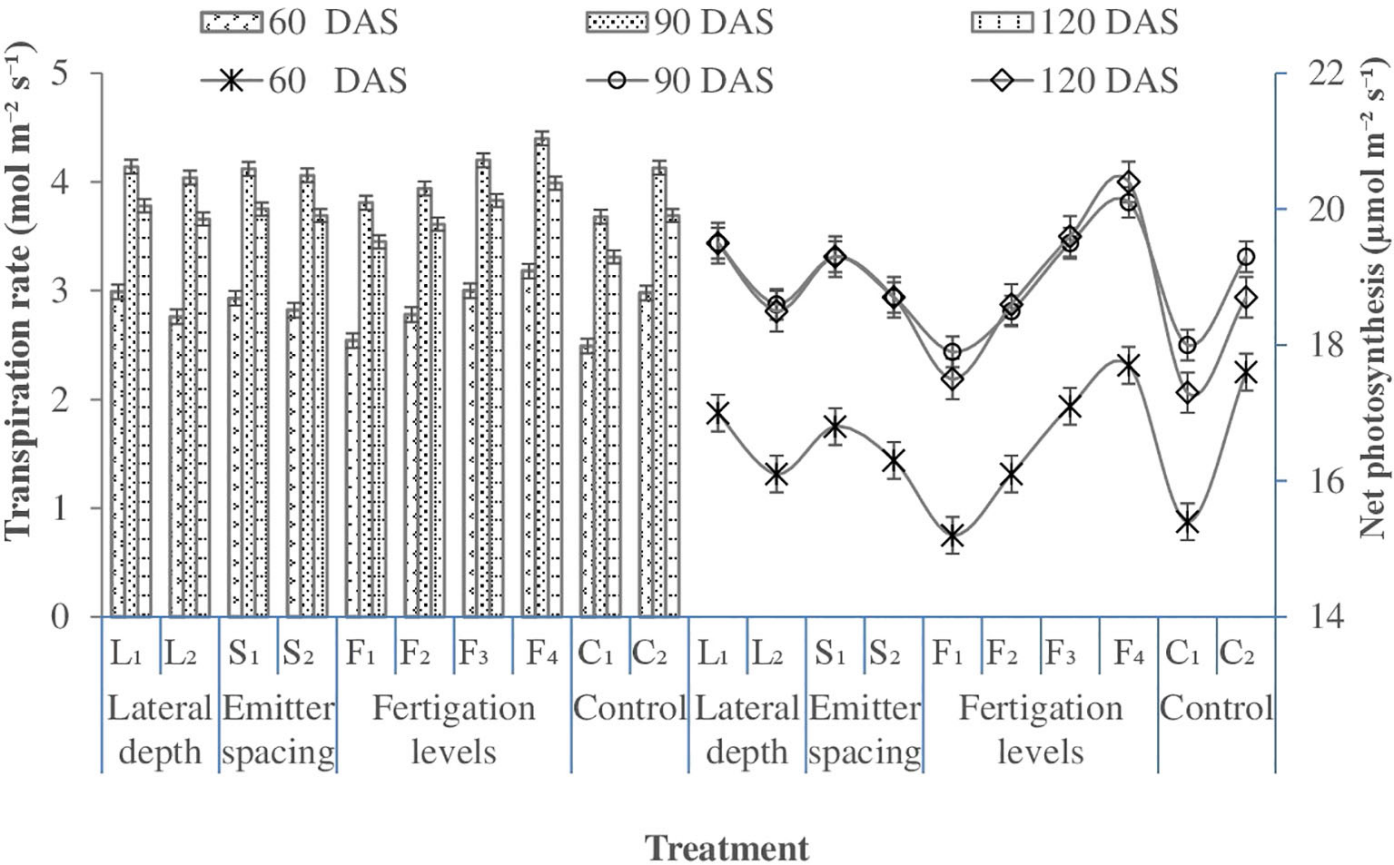
Effect of lateral depth, emitter spacing and fertigation scheduling on net photosynthesis rate of wheat (Pooled mean of 2 years). Where: L_1_, Lateral depth of 25 cm; L_2_, Lateral depth of 30 cm; S_1_, Emitter spacing of 30 cm; S_2_, Emitter spacing of 40 cm, F_1_ & F_2_, 100% N (112.5 kg N ha1) fertigation in 10 and 14 similar doses; F_3_ & F_4_: 125% N (140 kg N ha1) fertigation in 10 and 14 similar doses respectively; C_1_: Surface flood with 100% N (105 kg N ha‍1) through manual broadcasting in 2 similar doses (Control 1); C_2_: SDF at 20 cm depth with 100% N (112.5 kg N ha1) in 10 similar doses & emitter spacing at 20 cm (Control 2). For wheat: F_1_ & F_2_, 80% NP (100:50 kg NP ha1) fertigation in 8 and 10 similar doses respectively; F_3_ & F_4_, 100% NP (125:62.5 kg NP ha1) fertigation in 8 and 10 similar doses respectively; C_1_, Surface flood with 100% NP (125:62.5 kg NP ha1) through manual broadcasting in 2 similar doses (Control 1); C_2_, SDF at 20 cm depth with 100% N (100:50 kg NP ha ¨1) in 10 similar doses & emitter spacing at 20 cm (Control 2); SDF, Subsurface drip fertigation; DAS, days after sowing.

Nitrogen fertigation levels were the only factor exerting a significant influence on canopy temperature across all growth stages during both years (**Table 3**). Higher nitrogen application, particularly 125% N supplied in 10 and 14 equal splits (F3 and F4), consistently resulted in lower canopy temperature. Among control treatments, C2 exhibited significantly lower canopy temperature than surface flood (C1), with reductions of 5.6, 6.9, 8.9, and 13.0% at 60, 90, 120, and 150 DAS, respectively. The lower canopy temperature under SDF treatments may be attributed to enhanced transpiration rates (**Figure 6**) and improved canopy development, driven by efficient nutrient delivery in the root zone. In contrast, surface flood irrigation induced progressively greater moisture stress between irrigation events, leading to reduced transpiration, lower leaf conductance, and elevated canopy temperature, as reported by Gokila (2012).

#### 3.2.5 Chlorophyll content

Chlorophyll content (SPAD value) was highest during vegetative and early reproductive stages (60–120 DAS) and declined slightly towards maturity (150 DAS), reflecting progressive leaf senescence. Lateral placement depth and nitrogen fertigation levels significantly influenced SPAD values across all growth stages in both years, whereas emitter spacing had no significant effect (**Table 2**). Shallower lateral placement (25 cm) consistently resulted in higher chlorophyll content compared to deeper placement (30 cm), indicating improved nutrient and moisture availability in the active root zone. Nitrogen fertigation emerged as the dominant factor governing chlorophyll synthesis, with higher N supply producing progressively greater SPAD values across growth stages. The highest chlorophyll content was recorded under 125% N applied in 14 equal splits (F4), followed by 125% N in 10 splits (F3), while the lowest values were observed under 100% N applied in 10 splits (F1).Enhanced chlorophyll content under higher nitrogen supply reflects the central role of N in chlorophyll formation and photosynthetic activity, with peak values occurring during flowering and boll development stages, followed by a decline towards maturity. Similar trends have been reported by Nayyar et al. (1990) and Giri et al. (2016). Among control treatments, surface flood irrigation (C1) consistently recorded lower SPAD values than subsurface drip fertigation (C2), highlighting the superiority of SDF in maintaining leaf nitrogen status and photosynthetic capacity.

#### 3.2.6 Leaf area index

Pooled data indicated that shallower lateral placement (L1: 25 cm) significantly increased LAI compared to surface flood irrigation (C1), with increments of 35.3%, 30.4%, 21.1%, and 25.0% at 60, 90, 120, and 150 DAS, respectively (**Table 2**). Closer emitter spacing (S1: 30 cm) also enhanced LAI relative to wider spacing (S2: 40 cm), although both SDF treatments outperformed C1.Nitrogen fertigation levels markedly influenced LAI, with F4 (125% N) exhibiting higher values than F1 (100% N), showing increases of 26.3%, 18.5%, 16.7%, and 19.4% across the same growth stages. The elevated LAI under higher N supply is attributed to enhanced leaf production in indeterminate cotton, promoting canopy expansion. Frequent and split applications of N likely accelerated leaf area development, consistent with observations of Manickam and Pillai (2015).

The combination of L1 and S1 maximized LAI, surpassing C1 by 52.9%, 47.8%, 39.4%, and 42.9% at 60, 90, 120, and 150 DAS. Among controls, C2 consistently outperformed C1, highlighting the efficiency of SDF in supporting canopy development.

#### 3.2.7 Root length density

Root length density is a key indicator of cotton root development in response to soil moisture and nutrient availability. In 2021, lateral placement depth had minimal influence on RLD, except at 30–45 cm during 90–100 DAS, where shallow lateral placement (L1: 25 cm) exhibited slightly higher RLD (0.117 cm cm^-^³; **Supplementary Table 2**). In 2022, lateral depth significantly affected RLD, with L1 increasing RLD in the 0–30 cm layer by 4.6% relative to deeper placement (L2: 30 cm) during 90–100 DAS (**Supplementary Table 3**), consistent with Schenk and Jackson (2002) who reported enhanced upper root proliferation under shallower lateral spacings due to better soil and nutrient availability. Closer emitter spacing (S1: 30 cm) improved RLD by 7.4% (2021) and 7.1% (2022) at 90–100 DAS, and by 8.4% and 11.5% at maturity, respectively, in the 0–60 cm soil layer, compared to wider spacing (S2: 40 cm). This improvement is likely due to overlapping wetting zones providing uniform water and nutrient supply.

Nitrogen fertigation strongly influenced RLD, with both higher doses and increased split applications promoting root development. Fertigation with 125% N in 14 equal splits (F4) produced the highest RLD during both years (**Supplementary Tables 2, 3**). Relative to 100% N in 10 splits (F1), RLD under F4 increased by 15.9% and 23.0% (2021) and 24.7% and 31.7% (2022) at 90–100 DAS and maturity, respectively. These results underscore cotton root sensitivity to N availability, in agreement with Luo et al. (2015) and Chen et al. (2018a), who reported substantial RLD increases under moderate N fertilization. Enhanced root growth under F4 also reflects efficient carbon allocation from shoots to roots, facilitating root function and biomass accumulation (Chu et al., 2014). RLD decreased exponentially with soil depth, in consistency with Zuo et al. (2013). Among controls, C2 exhibited significantly higher total RLD than C1 (SFM) by 38.7–50.4% across 90–100 DAS and maturity stages in both years, corroborating findings of Sampathkumar et al. (2012), who observed greater root spread under drip irrigation compared to furrow irrigation. RLD at 15–30 cm was consistently lowest in C1 relative to SDF treatments, whereas interactive effects of lateral depth, emitter spacing, and N fertigation were not significant.

### 3.3 Physiological parameters of wheat

#### 3.3.1 Net photosynthesis

Net photosynthesis was higher under shallower lateral placement (L1: 25 cm), with deeper lateral placement (L2: 30 cm) causing a slight reduction of 5.9% in 2021–22 and 4.1% in 2022–23 at 60 DAS (**Figure 6**). The beneficial effect of shallow placement is attributed to uniform soil moisture and N availability near the root zone, enhancing photosynthetic activity (Waraich et al., 2011). However, as the crop progressed, lateral depth had no significant influence on Pn, suggesting that early-season mild moisture stress under deeper placement is mitigated as the root system develops. Emitter spacing could not affect Pn significantly. In contrast, N fertigation schedules exerted a marked effect, with higher Pn observed under F4 (100% NP in 10 splits), whereas the lowest Pn was evident under F1 (80% NP in 8 splits), declining by 16.5%, 12.3%, and 16.6% at 60, 90, and 120 DAS, respectively (**Figure 6**). The enhancement of Pn with increased NP fertigation can be attributed to direct nutrient supply, efficient translocation, increased leaf area, and higher chlorophyll content (**Table 4**), a finding consistent with Hamani et al. (2023), and Weih et al. (2016). These results indicate that optimal N management via fertigation positively regulates photosynthetic capacity and potentially improves wheat yield (Du et al., 2019).Among control treatments, C2 exhibited significantly higher Pn compared to C1 (SFM) at 60 DAS in 2021–22 and at 60 and 120 DAS in 2022–23. This trend aligns with Lawlor and Tezara (2009), who reported that soil moisture deficits reduce photosynthesis. In the present study, conventional flood irrigation (C1) caused greater temporal fluctuations in soil moisture, whereas SDF maintained more stable moisture levels, supporting higher Pn. Overall, all SDF treatments outperformed C1 in net photosynthesis.

**Table 4:** Effect of lateral depth, emitter spacing and fertigation scheduling on chlorophyll content and leaf area index of wheat (pooled mean of 2 years).

Lateral depth (L)	Emitter spacing (S)	Fertigation levels (F)	Chlorophyll content (SPAD value)			Leaf area index		
			60 DAS	90 DAS	120 DAS	60 DAS	90 DAS	120 DAS
L_1_ (25 cm)	S_1_ (30 cm)	F_1_	39.6	42.8	41.6	2.09	3.46	2.96
		F_2_	40.7	43.7	42.9	2.23	3.57	3.11
		F_3_	42.2	45.6	44.3	2.37	3.70	3.30
		F_4_	43.3	46.1	45.5	2.42	4.20	3.64
	S_2_ (40 cm)	F_1_	38.9	42.2	40.9	2.04	3.34	2.90
		F_2_	40.6	43	41.6	2.20	3.50	3.01
		F_3_	41.4	44.6	43.2	2.27	3.61	3.23
		F_4_	42.5	45.3	44.6	2.34	4.06	3.38
L_2_ (30 cm)	S_1_ (30 cm)	F_1_	38.5	41.4	40	2.03	3.26	2.78
		F_2_	39.4	42.3	41.2	2.14	3.41	2.93
		F_3_	41.0	44.0	42.4	2.20	3.58	3.16
		F_4_	42.0	44.8	42.9	2.28	3.90	3.32
	S_2_ (40 cm)	F_1_	37.4	40.8	39.0	1.99	3.10	2.72
		F_2_	38.4	42.0	40.4	2.07	3.34	2.83
		F_3_	39.9	42.7	42.4	2.14	3.51	3.08
		F_4_	41.0	43.3	42.6	2.27	3.77	3.28
L_1_			41.2	44.2	43	2.25	3.68	3.19
L_2_			39.7	42.7	41.4	2.14	3.49	3.01
S_1_			40.8	43.8	42.6	2.22	3.64	3.15
S_2_			40.0	43.0	41.8	2.16	3.53	3.05
F_1_			38.6	41.8	40.3	2.04	3.29	2.84
F_2_			39.8	42.8	41.5	2.16	3.46	2.97
F_3_			41.1	44.2	43.1	2.25	3.60	3.19
F_4_			42.2	44.9	43.9	2.33	3.98	3.40
C_1_			37.8	39.9	38.7	1.94	3.10	2.73
C_2_			40.6	43.8	42.0	2.21	3.41	3.00
LSD (p=0.05)								
Lateral depth (L)			0.5	1.0	1.1	NS	0.10	0.11
Emitter spacing (S)			NS	NS	NS	0.05	0.10	0.11
Fertigation levels (F)			0.9	1.2	1.2	0.07	0.10	0.10
Interactions			NS	NS	NS	NS	NS	NS
C_1_ vs C_2_			1.0	1.4	1.8	0.11	0.17	0.13
Treatments vs Controls			1.0	1.4	1.8	0.11	0.17	0.14

Where:

F_1_& F_2_, 80% NP (100:50 kg NP ha^-1^) fertigation in 8 and 10 similar doses respectively;

F_3_& F_4_, 100% NP (125:62.5 kg NP ha^-1^) fertigation in 8 and 10 similar doses respectively;

C_1_(SFM), Surface flood with 100% NP (125:62.5 kg NP ha^-1^) through manual broadcasting in 2 similar doses (Control 1);

C_2_, SDF at 20 cm depth with 100% N (100:50 kg NP ha^-1^) in 10 similar doses& emitter spacing at 20 cm (Control 2);

SDF, Subsurface drip fertigation;

DAS, days after sowing.

#### 3.3.2 Transpiration rate

Transpiration rate was higher under shallower lateral placement (L1: 25 cm), whereas deeper placement (L2: 30 cm) resulted in a significant reduction of 10.6% in 2021–22 and 7.7% in 2022–23 at 60 DAS (**Figure 6**). With crop advancement, lateral depth had only trivial effect on transpiration, indicating that early-season mild moisture stress under deeper placement is alleviated as the root system develops. Closer emitter spacing (30 cm, S1) enhanced transpiration rates (2.45 and 3.41 mol m^-^² s^-^¹) over wider spacing (40 cm, S2) at 60 DAS, reflecting better water availability in the root zone.

Fertigation schedules significantly influenced transpiration. Comparing F1 (80% NP in 8 splits) with F4 (100% NP in 10 splits) revealed reductions of 25.6–24.8% at 60 DAS, 17.4–14.3% at 90 DAS, and 22.2–11.1% at 120 DAS in 2021–22 and 2022–23, highlighting a strong relationship between nitrogen fertigation, net photosynthesis, and transpiration (Wang et al., 2023).Among control treatments, C2 exhibited 12.8–29.1%, 11.3–13.2%, and 9.1–14.7% higher transpiration at 60, 90, and 120 DAS, respectively, compared to C1 (SFM). Overall, all SDF treatments maintained higher transpiration rates than C1, indicating the advantage of SSD for improving canopy water use.

#### 3.3.3 Stomatal conductance

Stomatal conductance increased under shallower lateral placement (L1: 25 cm) compared to deeper placement (L2: 30 cm). Maximum values of 0.521, 0.506, and 0.471 mol m^-^² s^-^¹ at 60, 90, and 120 DAS, respectively, were recorded for L1 (pooled means), while emitter spacing could not exert significant effect at any stage (**Table 5**). Lower stomatal conductance under deeper lateral placement likely resulted from early-season water stress, which limited leaf area development. Fertigation levels significantly influenced stomatal conductance. Application of 100% NP in 10 equal splits (F4) resulted in the highest values of 0.492 and 0.550 mol m^-^² s^-^¹ at 60 DAS in 2021–22 and 2022–23, respectively. Enhanced N availability under F4 improved chlorophyll content, net photosynthesis, and canopy development, collectively contributing to higher stomatal conductance (Waraich et al., 2011). Among control treatments, C1 (SFM) exhibited reductions of 12.5%, 6.6%, and 16.3% at 60, 90, and 120 DAS, respectively, compared to C2. All SDF treatments consistently maintained higher stomatal conductance than C1, reflecting improved soil moisture distribution within the root zone (**Table 5**).

**Table 5:** Effect of lateral depth, emitter spacing and fertigation scheduling on stomatal conductance and canopy temperature of wheat (pooled mean of 2 years).

Lateral depth (L)	Emitter spacing (S)	Fertigation levels (F)	Stomatal conductance (mol m^-2^ s^-1^)			Canopy temperature (°C)			
			60 DAS	90 DAS	120 DAS	60 DAS	90 DAS	120 DAS	At harvest
L_1_ (25 cm)	S_1_ (30 cm)	F_1_	0.494	0.466	0.450	11.5	17.1	23.3	28.0
		F_2_	0.503	0.475	0.461	11.2	16.8	23.0	27.7
		F_3_	0.523	0.500	0.482	11.1	16.6	22.5	26.7
		F_4_	0.534	0.521	0.495	10.9	16.2	22.1	26.4
	S_2_ (40 cm)	F_1_	0.480	0.454	0.430	11.6	17.2	23.6	28.1
		F_2_	0.499	0.471	0.451	11.3	16.9	23.3	27.8
		F_3_	0.510	0.494	0.467	11.2	16.7	22.6	27.1
		F_4_	0.525	0.508	0.472	10.9	16.3	22.4	26.5
L_2_ (30 cm)	S_1_ (30 cm)	F_1_	0.449	0.443	0.419	11.6	17.2	23.7	28.1
		F_2_	0.474	0.462	0.446	11.4	16.9	23.4	27.9
		F_3_	0.499	0.488	0.453	11.2	16.7	22.8	27.3
		F_4_	0.517	0.501	0.465	11.1	16.5	22.5	26.6
	S_2_ (40 cm)	F_1_	0.434	0.439	0.409	11.8	17.5	23.8	28.3
		F_2_	0.478	0.451	0.439	11.6	17.2	23.5	28.0
		F_3_	0.494	0.475	0.452	11.5	16.8	23.1	27.4
		F_4_	0.509	0.499	0.453	11.1	16.6	22.8	26.7
L_1_			0.509	0.485	0.464	11.2	16.7	22.8	27.3
L_2_			0.482	0.469	0.442	11.4	16.9	23.2	27.5
S_1_			0.499	0.482	0.459	11.3	16.7	22.9	27.3
S_2_			0.491	0.473	0.447	11.4	17	23.1	27.4
F_1_			0.464	0.45	0.427	11.6	17.2	23.6	28.1
F_2_			0.488	0.465	0.450	11.4	17	23.3	27.8
F_3_			0.506	0.489	0.463	11.2	16.7	22.8	27.1
F_4_			0.521	0.506	0.471	11.0	16.4	22.4	26.5
C_1_			0.448	0.455	0.406	11.3	16.8	22.8	27.8
C_2_			0.504	0.485	0.472	11.1	16.5	22.3	27.0
CD (p=0.05)									
Lateral depth (L)			0.01	0.01	0.01	NS	0.2	0.3	NS
Emitter spacing (S)			NS	NS	NS	NS	NS	NS	NS
Fertigation levels (F)			0.01	0.02	0.01	0.3	0.3	0.4	0.4
Interactions			NS	NS	NS	NS	NS	NS	NS
C_1_ vs C_2_			0.03	0.02	0.02	NS	NS	NS	NS
Treatments vs Controls			0.03	NS	0.02	NS	NS	NS	NS

Where:

F_1_& F_2_, 80% NP (100:50 kg NP ha^-1^) fertigation in 8 and 10 similar doses respectively;

F_3_& F_4_, 100% NP (125:62.5 kg NP ha^-1^) fertigation in 8 and 10 similar doses respectively;

C_1_(SFM), Surface flood with 100% NP (125:62.5 kg NP ha^-1^) through manual broadcasting in 2 similar doses (Control 1);

C_2_, SDF at 20 cm depth with 100% N (100:50 kg NP ha^-1^) in 10 similar doses& emitter spacing at 20 cm (Control 2);

SDF, Subsurface drip fertigation;

DAS, days after sowing.

#### 3.3.4 Canopy temperature

Canopy temperature was significantly influenced only by fertigation levels (**Table 5**). Higher NP application, specifically 100% NP delivered in 8 and 10 equal splits (F3 and F4), resulted in lower canopy temperatures compared to lower NP levels (80% NP in F1 and F2). The reduction in canopy temperature under higher N supply, in the absence of water limitation, is consistent with previous findings (Mon et al., 2016).

#### 3.3.5 Chlorophyll content

Shallower lateral placement (L1: 25 cm) significantly enhanced chlorophyll content (SPAD) in wheat, whereas deeper placement (L2: 30 cm) led to mild early-season moisture and nutrient stress (**Table 4**). L1 increased SPAD by 4.1%, 3.6%, and 4.9% at 60, 90, and 120 DAS in 2021–22 and by 3.2% at 60 DAS in 2022-23. Fertigation levels also influenced chlorophyll, with 100% NP in 10 equal splits (F4) raising SPAD by 11.0%, 7.3%, and 9.9% compared to 80% NP in 8 splits (F1) in 2021-22, and by 7.9%, 7.3%, and 7.8% in the subsequent year. These responses reflect a close relationship between plant N status and SPAD, which also correlates well with leaf area index (Kubar et al., 2022). Similar trends of improved SPAD under higher N and optimized irrigation have been reported by Hamani et al. (2023). Among controls, C2 exhibited 7.5–9.8% higher chlorophyll than C1 (SFM), while all SDF treatments consistently outperformed C1.

#### 3.3.6 Leaf area index

Leaf area index was significantly influenced by lateral placement depth, emitter spacing, and fertigation schedules. Shallower lateral placement (L1: 25 cm) consistently produced higher LAI compared to deeper placement (L2: 30 cm), with reductions of 3.5%, 5.4%, and 6.9% at 60, 90, and 120 DAS in 2021-22, and 6.4%, 5.5%, and 4.8% in 2022-23, respectively. Closer emitter spacing (S1: 30 cm) enhanced LAI across growth stages, with pooled values of 2.22, 3.64, and 3.15 at 60, 90, and 120 DAS, respectively, compared to wider spacing (S2: 40 cm) which recorded 2.16, 3.53, and 3.05. The superior performance of shallow laterals and closer spacing is attributed to improved moisture and nutrient availability and overlapping wetting patterns (Kubar et al., 2022).

Fertigation schedules exerted a pronounced effect on LAI. Application of 100% NP in 10 equal splits (F4) consistently outperformed lower fertigation levels (F1: 80% NP in 8 splits), enhancing LAI by 14.2%, 20.9%, and 19.7% at 60, 90, and 120 DAS, respectively. This improvement corresponds with higher tiller production and reflects the positive influence of N on chlorophyll synthesis, cell size, and leaf expansion (Amanullah et al., 2007). Similar trends of increased LAI with higher fertigation have been reported under drip irrigation (Bai et al., 2019; Wang et al., 2023). Among control treatments, SFM (C1) exhibited the lowest LAI (1.94, 3.10, and 2.73 at 60, 90, and 120 DAS, respectively), averaging 9.9–13.9% lower than C2, clearly highlighting the benefits of SDF over surface flood irrigation (**Table 4**).

#### 3.3.7 Root length density

Root length density was largely unaffected by lateral placement depth and emitter spacing across both years. In contrast, fertigation schedules significantly influenced RLD in the upper 0–30 cm soil layer, whereas the deeper layer (30–60 cm) remained largely non-responsive (**Supplementary Tables 4, 5**). The application of 100% NP in 10 equal splits (F4) consistently improved RLD in the upper soil layers compared to lower fertigation levels. At 90–100 DAS and at maturity, 80% NP in 8 splits (F1) exhibited reductions of 9.9%, 4.9%, 11.7%, and 7.3% relative to F4, highlighting the importance of a robust root system for efficient N uptake in wheat. These results corroborate prior findings that nitrogen regulates RLD and facilitates dry matter translocation toward wheat spikes (Zhu et al., 2020; Wang et al., 2022). Appropriate N application enhances root morphological traits and increases the proportion of roots dedicated to nutrient absorption (Hao et al., 2009), while also stimulating enzymatic activities (Xu et al., 2015). Consequently, the higher RLD under F4 reflects improved root activity and nutrient uptake capacity.

Among control treatments, C2 exhibited significantly higher RLD in the upper 0–15 cm at maturity in 2021–22 and at 90–100 DAS in 2022–23 compared to C1 (SFM). These findings are consistent with Ma et al. (2022), who reported that surface drip fertigation promotes root growth in topsoil, while SDF (30 cm lateral depth) encourages deeper root development relative to flood irrigation.

### 3.4 Plant analysis

#### 3.4.1 N and P uptake by cotton

Nitrogen (N) and phosphorus (P) uptake by cotton varied significantly with fertigation levels, whereas lateral placement depth and emitter spacing had minimal effect (**Supplementary Table 6**). Uptake increased progressively from F1 to F4, with the highest values observed under 125% N applied in 14 equal splits (F4), reaching 265.2 kg ha^-^¹ in 2021 and 183.1 kg ha^-^¹ in 2022. This represented increases of 35.4% and 25.2%, respectively, over 100% N in 10 splits (F1). Phosphorus uptake followed a similar trend, with F4 recording 26.7 kg ha^-^¹ and 23.0 kg ha^-^¹ in 2021 and 2022, compared to the lowest values under F1 (24.3 and 17.5 kg ha^-^¹, respectively).Among control treatments, C1 (SFM) exhibited the lowest nutrient uptake, with N and P values of 172.0 and 23.5 kg ha^-1^ in 2021, and 134.3 and 15.9 kg ha^-^¹ in 2022. In contrast, C2 (SDF) demonstrated higher uptake, with 211.7 and 24.9 kg ha^-^¹ in 2021, and 160.1 and 22.5 kg ha^-^¹ in 2022. The superior nutrient uptake under higher fertigation levels and SDF treatments can be attributed to continuous water supply, which maintains soil moisture and nutrient availability, enhancing N and P absorption (Jayakumar et al., 2015).

The observed trends in N and P uptake closely matched dry matter accumulation, highlighting the dependence of nutrient uptake on both nutrient content and crop biomass (Constable et al., 1990). Additionally, higher available soil N and P (**Supplementary Table 6**) under increased fertigation further facilitated nutrient uptake. Similar findings were reported by Ayyadurai and Manickasundaram (2014), where 100% RDF via drip fertigation increased N and P uptake by 11–40% compared to reduced fertigation or conventional practices.

#### 3.4.2 N and P uptake by wheat

Nitrogen (N) and phosphorus (P) uptake by wheat was significantly influenced by fertigation levels, whereas lateral placement depth and emitter spacing had minimal impact (**Supplementary Table 6**). The highest nutrient uptake was observed under 100% NP delivered in 10 equal splits (F4), reaching 101.2 kg ha^-^¹ N and 14.1 kg ha^-^¹ P in 2021-22, and 109.6 kg ha^-^¹ N and 23.1 kg ha^-^¹ P in 2022-23. In contrast, lower fertigation (80% NP in 8 splits, F1) reduced N uptake by 27.8% and 27.6%, and P uptake by 18.5% and 30.5% in the respective years. Among control treatments, C1 (SFM) consistently exhibited the lowest nutrient uptake, with N values reduced by 21.8% and 13.2%, and P values of 10.9 and 13.5 kg ha^-^¹ in 2021–22 and 2022-23, respectively, compared to C2. The superior performance of SDF treatments can be attributed to efficient water and nutrient delivery, facilitating nutrient translocation from vegetative tissues to grain and ultimately enhancing wheat yield (Cao et al., 2017). Reduced uptake in C1 may be linked to intermittent water stress during critical stages such as tillering, jointing, and grain filling (Wang and Li, 2002). Comparable results were reported by Yan et al. (2019), where fertigation significantly improved N (2.06–2.33 times) and P (123%) uptake relative to surface flood irrigation without fertilization.

### 3.5 Yield

#### 3.5.1 Cotton yield attributes

##### 3.5.1.1 Bolls plant^-1^

Lateral placement depth significantly influenced boll formation in cotton. A shallower lateral at 25 cm (L1) produced 65.2 bolls plant^-^¹ compared to 55.1 bolls plant^-^¹ under the deeper lateral at 30 cm (L2). Closer emitter spacing (30 cm, S1) also enhanced boll count relative to wider spacing (40 cm, S2), yielding 63.3 and 57.0 bolls plant^-^¹, respectively (**Table 6**). Among fertigation treatments, 125% N applied in 14 equal doses (F4) recorded the highest bolls plant^-^¹, though values were comparable to 125% N in 10 equal splits (F3), whereas the lowest boll count (51.9) was observed under 100% N in 10 splits (F1). Across control treatments, C2 exhibited the maximum boll count (62.8), whereas C1 (SFM) had the minimum (50.0). These findings are consistent with earlier reports, where increased N application enhanced boll number (Patil and Janagoudar, 2016) and drip irrigation favored higher boll formation compared to furrow irrigation (Prajapati and Subbaiah, 2018).

**Table 6:** Effect of lateral depth, emitter spacing and fertigation scheduling on yield attributes and yield of cotton (pooled mean of 2 years).

Lateral depth (L)	Emitter spacing (S)	Fertigation levels (F)	`Yield attributes and yield of cotton				
			Bolls plant^-1^	Boll weight (g)	GOT (%)	Lint yield (kg ha^-1^)	Seed yield (kg ha^-1^)
L_1_ (25 cm)	S_1_ (30 cm)	F_1_	58.8	3.97	35.0	1136.7	2113.5
		F_2_	69.1	4.15	34.7	1217.9	2298.1
		F_3_	73.4	4.20	34.8	1308.3	2450.0
		F_4_	75.7	4.29	35.0	1353.4	2518.1
	S_2_ (40 cm)	F_1_	52.5	3.86	35.1	1024.4	1887.6
		F_2_	59.7	4.04	35.1	1136.0	2100.6
		F_3_	65.1	4.05	34.7	1196.1	2181.3
		F_4_	66.5	4.13	34.9	1209.3	2254.7
L_2_ (30 cm)	S_1_ (30 cm)	F_1_	49.6	3.82	35.3	980.5	1799.4
		F_2_	56.4	3.93	35.5	1058.4	1928.5
		F_3_	60.5	4.00	35.7	1127.6	2034.6
		F_4_	62.6	4.10	35.4	1155.0	2105.4
	S_2_ (40 cm)	F_1_	46.6	3.72	34.8	916.7	1722.3
		F_2_	51.6	3.86	35.2	991.5	1828.2
		F_3_	55.4	3.90	35.4	1031.8	1885.3
		F_4_	57.8	4.01	35.0	1096.7	2035.6
L_1_			65.2	4.08	34.9	1197.9	2221.4
L_2_			55.1	3.92	35.3	1044.8	1918.1
S_1_			63.3	6.06	35.2	1167.2	2154.4
S_2_			57.0	3.95	35.0	1082.6	1985.1
F_1_			51.9	3.84	35.0	1014.5	1876.2
F_2_			59.2	3.99	35.0	1101.0	2038.8
F_3_			63.6	4.03	35.1	1165.4	2136.1
F_4_			66.0	4.13	35.1	1203.6	2227.9
C_1_			50.0	3.73	34.3	881.2	1680.4
C_2_			62.8	4.11	34.9	1169.5	2178.4
LSD (p=0.05)							
Lateral depth (L)			2.3	0.07	NS	66.5	133.3
Emitter spacing (S)			2.3	0.07	NS	66.5	133.3
Fertigation levels (F)			3.4	0.09	NS	67.7	121.1
Interactions			NS	NS	NS	NS	NS
C_1_ vs C_2_			6.3	0.2	NS	104.1	179.0
Treatments vs Controls			NS	NS	0.7	106.6	183.3

Where:

F_1_& F_2_, 80% NP (100:50 kg NP ha^-1^) fertigation in 8 and 10 similar doses respectively;

F_3_& F_4_, 100% NP (125:62.5 kg NP ha^-1^) fertigation in 8 and 10 similar doses respectively;

C_1_(SFM), Surface flood with 100% NP (125:62.5 kg NP ha^-1^) through manual broadcasting in 2 similar doses (Control 1);

C_2_, SDF at 20 cm depth with 100% N (100:50 kg NP ha^-1^) in 10 similar doses& emitter spacing at 20 cm (Control 2);

SDF, Subsurface drip fertigation.

##### 3.5.1.2 Boll weight

Placement of laterals at 25cm deep (L_1_) and an emitter spacing of 30 cm (S_1_) resulted in the higher boll weight of 4.08 g and 4.06 g, respectively, outperforming the C_1_ (SFM) having least boll weight (3.73 g). Among the fertigation levels, (F_4_:125% N in 14 equal splits) recorded a boll weight of 4.13 g which outperformed both 100% N either in 10 (3.84 g) or 14 splits (3.99 g), while being on par with 125% N in 10 equal splits (4.03 g) in line with Kaur et al. (2025). Among control treatments, C_2_ exhibited the highest boll weight of 4.11 g (**Table 6**).

##### 3.5.1.3 Ginning out turn

Ginning out turn (GOT) refers to the quantity of lint in the seed cotton, and is a vital factor in determining the commercial value of the raw material (**Table 6**). Pooled data revealed higher GOT for L_2_ (35.3%), S_1_ (35.2%), and F_4_ (35.1%), which significantly outperformed the SFM (34.3) in consistency with Ali et al. (2017), who reported better GOT under drip (40.8%) as compared to furrow irrigation (39.3%).

##### 3.5.1.4 Lint yield

Lint yield was significantly influenced by lateral placement depth, emitter spacing, and N fertigation schedules (**Table 6**). A shallower lateral at 25 cm (L1) produced higher lint yield (1197.8 kg ha^-^¹) than the deeper lateral at 30 cm (L2, 1044.8 kg ha^-^¹), though L1 was comparable to C2. Closer emitter spacing (30 cm, S1) also enhanced yield (1167.2 kg ha^-^¹) relative to 40 cm spacing (S2, 1082.6 kg ha^-^¹), representing a 32.6% increase over C1 (SFM). Among fertigation levels, 125% N applied in 14 equal doses (F4) revealed the highest yield (1203.6 kg ha^-^¹), surpassing 100% N in 10 splits (F1) by 18.5% and C1 (SFM) by 36.4%, while F4, F3 (125% N in 10 splits, 1165.4 kg ha^-^¹), and C2 (1169.5 kg ha^-^¹) remained at par. These results indicate that optimal water and nutrient availability in the root zone under shallow lateral depth, closer emitter spacing, and higher N fertigation enhanced cotton productivity. Similar trends have been reported by Cetin and Kara (2019); Singh et al. (2018), and Neelakanth et al. (2019), who demonstrated superior yield under SDF compared to surface flood methods.

##### 3.5.1.5 Seed yield

Seed yield exhibited trends similar to lint yield across all treatments (**Table 6**). A shallower lateral at 25 cm (L1) produced the highest seed yield (2221.4 kg ha^-^¹), significantly surpassing the deeper lateral at 30 cm (L2, 1918.1 kg ha^-^¹), while remaining comparable to C2 (2178.4 kg ha^-^¹). Closer emitter spacing (30 cm, S1) also enhanced yield (2154.4 kg ha^-^¹) compared to 40 cm spacing (S2), representing an 8.5% increase and 28.2% higher than C1 (SFM). The combination of shallow lateral (L1) and closer emitter spacing (S1) resulted in a 32.2% improvement over C1.N fertigation levels significantly influenced seed yield. Maximum yield (2227.9 kg ha^-^¹) was recorded under 125% N in 14 equal splits (F4), though it was statistically at par with 125% N applied in 10 splits (F3, 2136.1 kg ha^-^¹). The lowest yield was noted with 100% N in 10 splits (F1, 1876.2 kg ha^-^¹). Among control treatments, C2 outperformed C1 (SFM), yielding 2178.4 kg ha^-^¹ and 1680.4 kg ha^-^¹, respectively. These findings corroborate earlier reports that optimal fertigation, shallow lateral depth, and closer emitter spacing enhance cotton productivity by improving water and nutrient availability in the root zone (Roopashree et al., 2016; Singh et al., 2022a).

#### 3.5.2 Wheat yield attributes

##### 3.5.2.1 Effective tillers

Deeper lateral placement at 30 cm (L2) significantly reduced effective tiller count compared to shallower placement at 25 cm (L1). Pooled means elucidated 402.0 m^-^² tillers under L1, representing a 10.5% increase over L2 (363.9 m^-^²), likely due to better topsoil moisture availability under shallower placement during critical wheat growth stages (**Table 7**). Closer emitter spacing (30 cm, S1) also enhanced tiller count (392.5 m^-^²) relative to wider spacing (40 cm, S2, 373.4 m^-^²).

**Table 7:** Effect of lateral depth, emitter spacing and fertigation scheduling on yield attributes and yield of wheat (pooled mean of 2 years).

Lateral depth (L)	Emitter spacing (S)	Fertigation levels (F)	`Yield attributes and grain yield of wheat				
			Effective tillers at harvest(m^-2^)	Ear length (cm)	Grains ear^-1^	1000 seed weight (g)	Grain yield (t ha^-1^)
L_1_ (25 cm)	S_1_ (30 cm)	F_1_	340.6	11.4	53.1	42.2	4.61
		F_2_	397.7	11.6	55.3	42.9	4.74
		F_3_	428.0	11.7	56.2	43.1	5.11
		F_4_	491.5	12.2	58.2	43.5	5.54
	S_2_ (40 cm)	F_1_	332.0	11.2	52.3	41.5	4.47
		F_2_	363.3	11.3	53.7	42.7	4.63
		F_3_	414.3	11.6	54.7	43.0	4.92
		F_4_	448.8	11.9	57.1	43.4	5.25
L_2_ (30 cm)	S_1_ (30 cm)	F_1_	324.8	11.2	51.8	41.6	4.25
		F_2_	346.5	11.3	53.0	42.3	4.51
		F_3_	393.2	11.5	54.1	43.0	4.80
		F_4_	417.8	11.9	56.6	43.2	5.00
	S_2_ (40 cm)	F_1_	299.6	10.8	51.1	40.9	4.07
		F_2_	338.3	11.2	51.7	42.1	4.35
		F_3_	384.1	11.4	54.1	42.7	4.61
		F_4_	406.7	11.7	55.8	43.1	4.89
L_1_			402.0	11.6	55.1	42.8	4.91
L_2_			363.9	11.4	53.5	42.4	4.56
S_1_			392.5	11.6	54.8	42.7	4.82
S_2_			373.4	11.4	53.8	42.4	4.65
F_1_			324.3	11.1	52.1	41.5	4.35
F_2_			361.4	11.3	53.4	42.5	4.56
F_3_			404.9	11.5	54.8	42.9	4.86
F_4_			441.2	11.9	56.9	43.3	5.17
C_1_			303.6	11.0	50.8	40.6	4.32
C_2_			427.5	11.4	53.2	42.9	5.00
LSD (p=0.05)							
Lateral depth (L)			6.5	NS	1.3	NS	0.11
Emitter spacing (S)			6.5	NS	1.3	NS	0.11
Fertigation levels (F)			13.6	0.3	1.3	0.8	0.24
Interactions			NS	NS	NS	NS	NS
C_1_ vs C_2_			10.3	NS	1.8	1.1	0.34
Treatments vs Controls			10.6	NS	1.9	1.1	NS

Where:

F_1_& F_2_, 80% NP (100:50 kg NP ha^-1^) fertigation in 8 and 10 similar doses respectively;

F_3_& F_4_, 100% NP (125:62.5 kg NP ha^-1^) fertigation in 8 and 10 similar doses respectively;.

C_1_(SFM), Surface flood with 100% NP (125:62.5 kg NP ha^-1^) through manual broadcasting in 2 similar doses (Control 1);

C_2_, SDF at 20 cm depth with 100% N (100:50 kg NP ha^-1^) in 10 similar doses& emitter spacing at 20 cm (Control 2);

SDF, Subsurface drip fertigation.

Fertigation schedules markedly influenced effective tillers. Application of 100% NP in 10 equal splits (F4) resulted in the highest count (441.2 m^-^²), a 36.0% increase over 80% NP in 8 splits (F1). These results align with previous studies reporting improved effective tillers under split fertigation (Abdelraouf et al., 2013). Split application during early irrigations was found more effective than conventional top-dressing in wheat (Alam et al., 2005).Among control treatments, C1 (SFM) exhibited the least tiller count, with reductions of 32.4%, 29.3%, and 40.8% compared to L1, S1, and C2, respectively. Improved tiller development under SDF can be attributed to precise nutrient timing and uniform distribution, which enhanced photosynthesis, chlorophyll content, root growth, and leaf area index, collectively promoting higher effective tiller count (Rao et al., 2016).

##### 3.5.2.2 Ear length and grains ear^-1^

Ear length and grains ear^-^¹ were largely unaffected by lateral placement depth and emitter spacing (**Table 7**). However, fertigation levels significantly influenced these yield attributes. Reduced fertigation (80% NP in 8 equal splits, F1) led to lower ear length and grains ear^-^¹, while the highest values were observed under 100% NP applied in 10 equal splits (F4), with ear length of 11.9 cm and 56.9 grains ear^-^¹. Correspondingly, the lowest ear length (11.0 cm) and grains ear^-^¹ (50.8) were recorded under C1 (SFM).Compared to F4, F1 exhibited a 7.0% decrease in ear length and 9.0% reduction in grains ear^-^¹, whereas C1 recorded a 12.0% reduction. These results indicate that split fertigation through SDF improves ear development and grain formation relative to conventional irrigation (SFM). Similar trends have been reported by Karangiya et al. (2019), where drip fertigation at 100% RDF (120:60:60 kg NPK ha^-^¹) increased ear length (8.13 cm) compared to 40% RDF (6.40 cm), and by Malve et al. (2017), who observed higher grains ear^-^¹ (33.5) with 160 kg N ha^-^¹ relative to 80 kg N ha^-^¹ (30.2) under drip irrigation.

##### 3.5.2.3 Test weight

The 1000-grain weight of wheat was significantly influenced only by NP fertigation schedules, while lateral depth and emitter spacing had no effect (**Table 7**). Among fertigation levels, 100% NP application produced the highest 1000-grain weight (43.3 g), compared to 80% NP (41.5 g), representing a 4.3% increase when shifting from F1 (80% NP, 8 splits) to F4 (100% NP, 14 splits). The lowest 1000-grain weight was observed under C1 (SFM, 40.6 g), showing a reduction of 5.7% and 6.7% relative to C2 and F4, respectively. The increase in grain weight under higher fertigation levels is attributable to improved physiological parameters that enhance grain filling, consistent with findings of Frederick et al. (2001), who reported superior test weight under SDF compared to conventional irrigation.

##### 3.5.2.4 Wheat grain yield

Grain yield of wheat was negatively affected by increased lateral placement depth and wider emitter spacing, whereas higher NP fertigation levels exerted a positive influence (**Table 7**). Shallow lateral placement at 25 cm (L1) produced 4.91 t ha^-^¹, while deeper placement at 30 cm (L2) reduced yield by 7.7% to 4.56 t ha^-^¹, likely due to restricted nutrient and water availability near the root zone during early crop growth (Ali et al., 2007; Sidhu et al., 2019). Closer emitter spacing (30 cm, S1) recorded 4.82 t ha^-^¹, 3.7% higher than 40 cm spacing (S2, 4.65 t ha^-^¹), reflecting improved soil moisture distribution and reduced stress for plants (Chen et al., 2015).

Among fertigation schedules, 100% NP applied in 10 equal splits (F4) achieved the highest grain yield (5.1 t ha^-^¹), associated with superior physiological and growth traits including higher chlorophyll content, RLD, effective tillers, LAI, ear length, and grains ear^-^¹ (**Tables 5**, **4**). Frequent and uniform NP delivery near the root zone promoted vigorous root and shoot growth, enhanced stomatal conductance, and improved grain-filling, thereby increasing yield (Abad et al., 2004; Singh et al., 2011). Among control treatments, C2 outperformed C1 (SFM), with the latter showing a 15.8% reduction in grain yield. These results highlight the advantage of SDF in providing optimized nutrient supply and consistent soil moisture, enhancing physiological performance and yield attributes compared to conventional irrigation, consistent with previous reports (Tasal and Pawar, 2015; Suradkar et al., 2020).

## 4 Conclusion

Water–nutrient interactions under reduced tillage remain poorly understood in intensive cotton–wheat systems, despite their relevance to water-scarce agroecosystems. The present study demonstrated that subsurface drip fertigation (SDF), when combined with reduced tillage can significantly enhance the productivity of the cotton–wheat cropping system (CWCS) in low-tilled environments by improving soil physical properties and crop physiological parameters. In contrast, conventional surface flood irrigation (SFM) led to reduced crop performance, driven by poor soil attributes, including lower infiltration rates, higher bulk density, and consequent reduction in reproductive traits. For cotton, a lateral depth of 25 cm and closer emitter spacing of 30 cm improved lint yield, which was 32.6% higher than SFM. Fertigation with 125% N in 14 equal splits (F4) further enhanced lint yield, surpassing 100% N in 10 splits (F3) by 18.5% and SFM by 36.4%. In wheat, shallow lateral placement (25 cm) and closer emitter spacing (30 cm) increased grain yield, while deeper laterals (30 cm) and wider emitter spacing (40 cm) reduced yield. Fertigation with 100% NP in 10 splits (F4) maximized wheat productivity. SFM (C1) consistently resulted in lower yields, with a 15.8% reduction in wheat grain yield compared to SDF. Overall, conventional tillage and SFM represent major constraints for sustaining CWCS productivity in north-western India. Adoption of reduced tillage combined with SDF improved soil bulk density, infiltration, and soil organic carbon, while enhancing physiological traits and yield. The optimal combination i.e., a lateral depth of 25 cm, emitter spacing of 30 cm, and fertigation of 125% N (140 kg N ha^-1^) in 14 splits for cotton and 100% NP (125-62.5 kg NP ha^-1^) in 10 splits for wheat provided superior yield and yield attributes by promoting better soil–plant interactions and resource use efficiency.

## Data Availability

The original contributions presented in the study are included in the article/**Supplementary Material**. Further inquiries can be directed to the corresponding author.
