# Supplementary material for: Subsurface fertigation modifies soil–plant–water interactions to improve productivity of cotton–wheat systems under reduced tillage
Source: Front Plant Sci. 2026 Mar 26;17:1813404. doi: 10.3389/fpls.2026.1813404 (PMC13061729; doi:10.3389/fpls.2026.1813404)
Supplement: Supplementary file 1 [file Table1.docx]

Supplementary Table 1 Crop-specific irrigation scheduling for cotton and wheat during crop seasons

| Irrigation scheduling dates | | | | |
| --- | --- | --- | --- | --- |
|  | Cotton | | Wheat | |
| S. no. | 2021 | 2022 | 2021-22 | 2022-23 |
| Subsurface drip irrigation (SDF) | | | | |
| 1 | 03.06.2021 | 08.06.2022 | 10.12.2021 | 07.12.2022 |
| 2 | 09.06.2021 | 15.06.2022 | 21.12.2021 | 13.12.2022 |
| 3 | 15.06.2021 | 29.06.2022 | 27.12.2021 | 20.12.2022 |
| 4 | 20.06.2021 | 05.07.2022 | 14.02.2022 | 28.12.2022 |
| 5 | 29.06.2021 | 12.07.2022 | 24.02.2022 | 04.01.2023 |
| 6 | 06.07.2021 | 29.07.2022 | 03.03.2022 | 10.01.2023 |
| 7 | 13.07.2021 | 17.08.2022 | 09.03.2022 | 17.01.2023 |
| 8 | 20.07.2021 | 23.08.2022 | 16.03.2022 | 24.01.2023 |
| 9 | 27.07.2021 | 30.08.2022 | 23.03.2022 | 31.01.2023 |
| 10 | 16.08.2021 | 07.09.2022 | 30.03.2022 | 07.02.2023 |
| 11 | 23.08.2021 | 14.09.2022 | 06.04.2022 | 13.02.2023 |
| 12 | 29.08.2021 | 21.09.2022 |  | 21.02.2023 |
| 13 | 09.09.2021 | 03.10.2022 |  | 01.03.2023 |
| 14 | 20.09.2021 |  |  | 07.03.2023 |
| 15 |  |  |  | 13.03.2023 |
| Surface Flood irrigation (SFM) | | | | |
| 1 | 02.06.2021 | 08.06.2022 | 10.12.2021 | 13.12.2022 |
| 2 | 29.06.2021 | 09.07.2022 | 14.02.2021 | 17.01.2023 |
| 3 | 20.07.2021 | 17.08.2022 | 09.03.2021 | 07.02.2023 |
| 4  5 | 31.08.2021  25.09.2021 | 07.09.2022  03.10.2022 | 06.04.2021 | 07.03.2023 |

Supplementary Table 2 Effect of lateral depth, emitter spacing and fertigation scheduling on root length density of cotton during 2021

| Lateral  depth (L) | Emitter spacing (S) | Fertigation levels (F) | Root length density (cm cm^-3^) | | | | | | | | | |
| --- | --- | --- | --- | --- | --- | --- | --- | --- | --- | --- | --- | --- |
|  |  |  | 90-100 DAS | | | |  | at maturity | | | | |
|  |  |  | 0- 15 cm | 15-30 cm | 30-45 cm | 45-60 cm |  | 0- 15 cm | 15-30 cm | 30-45 cm | 45-60 cm |  |
| L_1_  (25 cm) | S_1_  (30 cm) | F_1_ | 0.360 | 0.180 | 0.103 | 0.070 |  | 0.390 | 0.227 | 0.140 | 0.093 |  |
|  |  | F_2_ | 0.370 | 0.187 | 0.110 | 0.077 |  | 0.403 | 0.240 | 0.147 | 0.103 |  |
|  |  | F_3_ | 0.397 | 0.213 | 0.130 | 0.090 |  | 0.413 | 0.263 | 0.160 | 0.107 |  |
|  |  | F_4_ | 0.413 | 0.240 | 0.150 | 0.107 |  | 0.450 | 0.277 | 0.177 | 0.127 |  |
|  | S_2_  (40 cm) | F_1_ | 0.340 | 0.163 | 0.093 | 0.063 |  | 0.370 | 0.203 | 0.107 | 0.080 |  |
|  |  | F_2_ | 0.357 | 0.173 | 0.100 | 0.073 |  | 0.390 | 0.233 | 0.127 | 0.090 |  |
|  |  | F_3_ | 0.370 | 0.190 | 0.120 | 0.093 |  | 0.403 | 0.253 | 0.137 | 0.120 |  |
|  |  | F_4_ | 0.390 | 0.210 | 0.127 | 0.100 |  | 0.417 | 0.257 | 0.160 | 0.097 |  |
| L_2_  (30 cm) | S_1_  (30 cm) | F_1_ | 0.353 | 0.170 | 0.090 | 0.070 |  | 0.387 | 0.217 | 0.120 | 0.090 |  |
|  |  | F_2_ | 0.360 | 0.187 | 0.097 | 0.077 |  | 0.400 | 0.240 | 0.130 | 0.100 |  |
|  |  | F_3_ | 0.383 | 0.203 | 0.140 | 0.090 |  | 0.417 | 0.260 | 0.150 | 0.123 |  |
|  |  | F_4_ | 0.393 | 0.220 | 0.140 | 0.100 |  | 0.443 | 0.270 | 0.170 | 0.137 |  |
|  | S_2_  (40 cm) | F_1_ | 0.333 | 0.153 | 0.083 | 0.063 |  | 0.347 | 0.207 | 0.100 | 0.080 |  |
|  |  | F_2_ | 0.350 | 0.170 | 0.100 | 0.067 |  | 0.367 | 0.220 | 0.120 | 0.090 |  |
|  |  | F_3_ | 0.370 | 0.187 | 0.110 | 0.087 |  | 0.413 | 0.230 | 0.153 | 0.117 |  |
|  |  | F_4_ | 0.380 | 0.203 | 0.127 | 0.093 |  | 0.410 | 0.240 | 0.157 | 0.103 |  |
| L_1_ | | | 0.375 | 0.195 | 0.117 | 0.084 |  | 0.405 | 0.244 | 0.144 | 0.102 |  |
| L_2_ | | | 0.365 | 0.187 | 0.111 | 0.081 |  | 0.399 | 0.235 | 0.138 | 0.105 |  |
| S_1_ | | | 0.379 | 0.200 | 0.12 | 0.085 |  | 0.413 | 0.249 | 0.149 | 0.110 |  |
| S_2_ | | | 0.361 | 0.181 | 0.108 | 0.080 |  | 0.390 | 0.230 | 0.133 | 0.097 |  |
| F_1_ | | | 0.347 | 0.167 | 0.092 | 0.067 |  | 0.374 | 0.214 | 0.117 | 0.086 |  |
| F_2_ | | | 0.359 | 0.179 | 0.102 | 0.074 |  | 0.390 | 0.233 | 0.131 | 0.096 |  |
| F_3_ | | | 0.38 | 0.198 | 0.125 | 0.09 |  | 0.412 | 0.252 | 0.150 | 0.117 |  |
| F_4_ | | | 0.394 | 0.218 | 0.136 | 0.100 |  | 0.430 | 0.261 | 0.166 | 0.116 |  |
| C_1_ | | | 0.34 | 0.140 | 0.080 | 0.060 |  | 0.370 | 0.177 | 0.077 | 0.073 |  |
| C_2_ | | | 0.393 | 0.230 | 0.140 | 0.097 |  | 0.442 | 0.270 | 0.150 | 0.120 |  |
| CD (p=0.05) | | |  |  |  |  |  |  |  |  |  |  |
| Lateral depth (L) | | |  |  |  |  |  |  |  |  |  |  |
| Emitter spacing (S) | | | NS | NS | 0.004 | NS |  | NS | NS | NS | NS |  |
| Fertigation levels (F) | | | 0.01 | 0.01 | 0.004 | NS |  | 0.01 | 0.01 | 0.01 | 0.01 |  |
| Interactions | | | 0.01 | 0.01 | 0.001 | 0.01 |  | 0.02 | 0.01 | 0.01 | 0.02 |  |
| C_1_ vs C_2_ | | | NS | NS | NS | NS |  | NS | NS | NS | NS |  |
| Treatments vs Controls | | | 0.02 | 0.01 | 0.01 | 0.02 |  | 0.02 | 0.02 | 0.01 | 0.02 |  |

Where: -

F_1_& F_2_: 100% N (112.5 kg N ha^-1^) fertigation in 10 and 14 similar doses;

F_3_& F_4_: 125% N (140 kg N ha^-1^) fertigation in 10 and 14 similar doses respectively;

C_1_(SFM):Surface flood with 100% N (105 kg N ha^‑1^) through manual broadcasting in 2 similar doses (Control 1);

C_2_: SDF at 20 cm depth with 100% N (112.5 kg N ha^-1^) in 10 similar doses& emitter spacing at 20 cm (Control 2);

SDF: Subsurface drip fertigation;

DAS: days after sowing

Supplementary Table 3 Effect of lateral depth, emitter spacing and fertigation scheduling on root length density of cotton during 2022

| Lateral  depth (L) | Emitter spacing (S) | Fertigation levels (F) | Root length density (cm cm^-3^) | | | | | | | | | |
| --- | --- | --- | --- | --- | --- | --- | --- | --- | --- | --- | --- | --- |
|  |  |  | 90-100 DAS | | | |  | at maturity | | | | |
|  |  |  | 0- 15 cm | 15-30 cm | 30-45 cm | 45-60 cm |  | 0- 15 cm | 15-30 cm | 30-45 cm | 45-60 cm |  |
| L_1_  (25 cm) | S_1_  (30 cm) | F_1_ | 0.343 | 0.167 | 0.083 | 0.057 |  | 0.280 | 0.107 | 0.070 | 0.060 |  |
|  |  | F_2_ | 0.330 | 0.177 | 0.103 | 0.070 |  | 0.297 | 0.123 | 0.077 | 0.063 |  |
|  |  | F_3_ | 0.353 | 0.180 | 0.130 | 0.083 |  | 0.310 | 0.133 | 0.090 | 0.073 |  |
|  |  | F_4_ | 0.373 | 0.190 | 0.140 | 0.103 |  | 0.327 | 0.160 | 0.097 | 0.097 |  |
|  | S_2_  (40 cm) | F_1_ | 0.307 | 0.127 | 0.063 | 0.060 |  | 0.263 | 0.080 | 0.050 | 0.043 |  |
|  |  | F_2_ | 0.323 | 0.143 | 0.080 | 0.070 |  | 0.283 | 0.113 | 0.063 | 0.047 |  |
|  |  | F_3_ | 0.333 | 0.150 | 0.113 | 0.070 |  | 0.290 | 0.127 | 0.070 | 0.070 |  |
|  |  | F_4_ | 0.347 | 0.173 | 0.130 | 0.087 |  | 0.313 | 0.140 | 0.083 | 0.063 |  |
| L_2_  (30 cm) | S_1_  (30 cm) | F_1_ | 0.297 | 0.160 | 0.080 | 0.063 |  | 0.270 | 0.120 | 0.067 | 0.050 |  |
|  |  | F_2_ | 0.30 | 0.140 | 0.100 | 0.070 |  | 0.287 | 0.130 | 0.073 | 0.060 |  |
|  |  | F_3_ | 0.340 | 0.160 | 0.117 | 0.077 |  | 0.313 | 0.140 | 0.077 | 0.090 |  |
|  |  | F_4_ | 0.330 | 0.180 | 0.130 | 0.087 |  | 0.323 | 0.143 | 0.087 | 0.077 |  |
|  | S_2_  (40 cm) | F_1_ | 0.303 | 0.133 | 0.067 | 0.070 |  | 0.260 | 0.090 | 0.047 | 0.047 |  |
|  |  | F_2_ | 0.317 | 0.153 | 0.080 | 0.070 |  | 0.280 | 0.120 | 0.050 | 0.057 |  |
|  |  | F_3_ | 0.330 | 0.173 | 0.107 | 0.077 |  | 0.287 | 0.127 | 0.060 | 0.080 |  |
|  |  | F_4_ | 0.323 | 0.180 | 0.117 | 0.083 |  | 0.307 | 0.140 | 0.067 | 0.080 |  |
| L_1_ | | | 0.339 | 0.163 | 0.105 | 0.080 |  | 0.295 | 0.123 | 0.075 | 0.065 |  |
| L_2_ | | | 0.320 | 0.160 | 0.100 | 0.075 |  | 0.291 | 0.126 | 0.066 | 0.068 |  |
| S_1_ | | | 0.336 | 0.169 | 0.110 | 0.076 |  | 0.301 | 0.132 | 0.080 | 0.071 |  |
| S_2_ | | | 0.323 | 0.154 | 0.095 | 0.073 |  | 0.285 | 0.117 | 0.061 | 0.061 |  |
| F_1_ | | | 0.313 | 0.147 | 0.073 | 0.063 |  | 0.268 | 0.099 | 0.059 | 0.050 |  |
| F_2_ | | | 0.323 | 0.153 | 0.091 | 0.070 |  | 0.287 | 0.122 | 0.066 | 0.057 |  |
| F_3_ | | | 0.339 | 0.166 | 0.117 | 0.077 |  | 0.300 | 0.132 | 0.074 | 0.078 |  |
| F_4_ | | | 0.343 | 0.181 | 0.129 | 0.090 |  | 0.318 | 0.146 | 0.084 | 0.079 |  |
| C_1_ | | | 0.273 | 0.120 | 0.057 | 0.040 |  | 0.283 | 0.090 | 0.048 | 0.047 |  |
| C_2_ | | | 0.357 | 0.183 | 0.117 | 0.080 |  | 0.313 | 0.123 | 0.094 | 0.101 |  |
| CD (p=0.05) | | |  |  |  |  |  |  |  |  |  |  |
| Lateral depth (L) | | | 0.01 | 0.01 | NS | NS |  | NS | NS | 0.01 | NS |  |
| Emitter spacing (S) | | | 0.01 | 0.02 | 0.01 | NS |  | 0.009 | 0.01 | 0.01 | 0.01 |  |
| Fertigation levels (F) | | | 0.02 | 0.01 | 0.01 | 0.01 |  | 0.01 | 0.01 | 0.01 | 0.01 |  |
| Interactions | | | NS | NS | NS | NS |  | NS | NS | NS | NS |  |
| C_1_ vs C_2_ | | | 0.03 | 0.03 | 0.03 | 0.03 |  | 0.02 | 0.02 | 0.01 | 0.01 |  |
| Treatments vs Controls | | | NS | NS | NS | NS |  | NS | 0.02 | NS | NS |  |

Where: -

F_1_& F_2_: 100% N (112.5 kg N ha^-1^) fertigation in 10 and 14 similar doses;

F_3_& F_4_: 125% N (140 kg N ha^-1^) fertigation in 10 and 14 similar doses respectively;

C_1_(SFM):Surface flood with 100% N (105 kg N ha^‑1^) through manual broadcasting in 2 similar doses (Control 1);

C_2_: SDF at 20 cm depth with 100% N (112.5 kg N ha^-1^) in 10 similar doses& emitter spacing at 20 cm (Control 2);

SDF: Subsurface drip fertigation;

DAS: days after sowing

Supplementary Table 4 Effect of lateral depth, emitter spacing and fertigation scheduling on root length density of wheat during 2021-22

| Lateral  depth (L) | Emitter spacing (S) | Fertigation levels (F) | Root length density (cm cm^-3^) | | | | | | | | | |
| --- | --- | --- | --- | --- | --- | --- | --- | --- | --- | --- | --- | --- |
|  |  |  | 90-100 DAS | | | |  | at maturity | | | | |
|  |  |  | 0- 15 cm | 15-30 cm | 30-45 cm | 45-60 cm |  | 0- 15 cm | 15-30 cm | 30-45 cm | 45-60 cm |  |
| L_1_  (25 cm) | S_1_  (30 cm) | F_1_ | 1.070 | 0.737 | 0.447 | 0.220 |  | 0.823 | 0.507 | 0.357 | 0.147 |  |
|  |  | F_2_ | 1.107 | 0.767 | 0.450 | 0.227 |  | 0.823 | 0.513 | 0.360 | 0.153 |  |
|  |  | F_3_ | 1.127 | 0.797 | 0.457 | 0.233 |  | 0.833 | 0.517 | 0.373 | 0.157 |  |
|  |  | F_4_ | 1.203 | 0.823 | 0.457 | 0.233 |  | 0.860 | 0.550 | 0.377 | 0.157 |  |
|  | S_2_  (40 cm) | F_1_ | 1.030 | 0.727 | 0.437 | 0.217 |  | 0.813 | 0.503 | 0.350 | 0.143 |  |
|  |  | F_2_ | 1.097 | 0.757 | 0.443 | 0.223 |  | 0.820 | 0.517 | 0.360 | 0.150 |  |
|  |  | F_3_ | 1.113 | 0.767 | 0.450 | 0.230 |  | 0.830 | 0.517 | 0.367 | 0.150 |  |
|  |  | F_4_ | 1.153 | 0.787 | 0.457 | 0.230 |  | 0.847 | 0.520 | 0.370 | 0.153 |  |
| L_2_  (30 cm) | S_1_  (30 cm) | F_1_ | 1.070 | 0.723 | 0.437 | 0.217 |  | 0.810 | 0.500 | 0.350 | 0.143 |  |
|  |  | F_2_ | 1.080 | 0.760 | 0.440 | 0.223 |  | 0.830 | 0.513 | 0.357 | 0.147 |  |
|  |  | F_3_ | 1.120 | 0.767 | 0.447 | 0.227 |  | 0.837 | 0.517 | 0.370 | 0.150 |  |
|  |  | F_4_ | 1.153 | 0.783 | 0.450 | 0.227 |  | 0.843 | 0.533 | 0.377 | 0.150 |  |
|  | S_2_  (40 cm) | F_1_ | 1.053 | 0.707 | 0.423 | 0.213 |  | 0.803 | 0.497 | 0.340 | 0.140 |  |
|  |  | F_2_ | 1.073 | 0.733 | 0.443 | 0.217 |  | 0.823 | 0.507 | 0.357 | 0.140 |  |
|  |  | F_3_ | 1.107 | 0.767 | 0.447 | 0.227 |  | 0.827 | 0.510 | 0.363 | 0.147 |  |
|  |  | F_4_ | 1.147 | 0.773 | 0.450 | 0.230 |  | 0.840 | 0.520 | 0.370 | 0.150 |  |
| L_1_ | | | 1.113 | 0.77 | 0.45 | 0.227 |  | 0.831 | 0.519 | 0.364 | 0.151 |  |
| L_2_ | | | 1.100 | 0.752 | 0.442 | 0.223 |  | 0.827 | 0.512 | 0.36 | 0.146 |  |
| S_1_ | | | 1.116 | 0.769 | 0.448 | 0.226 |  | 0.833 | 0.518 | 0.365 | 0.150 |  |
| S_2_ | | | 1.097 | 0.752 | 0.444 | 0.222 |  | 0.825 | 0.511 | 0.359 | 0.147 |  |
| F_1_ | | | 1.056 | 0.724 | 0.436 | 0.217 |  | 0.812 | 0.502 | 0.349 | 0.143 |  |
| F_2_ | | | 1.089 | 0.754 | 0.444 | 0.223 |  | 0.824 | 0.513 | 0.359 | 0.148 |  |
| F_3_ | | | 1.117 | 0.775 | 0.450 | 0.229 |  | 0.832 | 0.515 | 0.368 | 0.151 |  |
| F_4_ | | | 1.164 | 0.792 | 0.454 | 0.230 |  | 0.848 | 0.531 | 0.374 | 0.153 |  |
| C_1_ | | | 1.063 | 0.698 | 0.420 | 0.212 |  | 0.795 | 0.489 | 0.352 | 0.133 |  |
| C_2_ | | | 1.105 | 0.742 | 0.440 | 0.224 |  | 0.838 | 0.511 | 0.363 | 0.149 |  |
| CD (p=0.05) | | |  |  |  |  |  |  |  |  |  |  |
| Lateral depth (L) | | | NS | NS | NS | NS |  | NS | NS | NS | NS |  |
| Emitter spacing (S) | | | NS | NS | NS | NS |  | NS | NS | NS | NS |  |
| Fertigation levels (F) | | | 0.07 | 0.04 | NS | NS |  | 0.02 | NS | NS | NS |  |
| Interactions | | | NS | NS | NS | NS |  | NS | NS | NS | NS |  |
| C_1_ vs C_2_ | | | NS | NS | NS | NS |  | 0.02 | NS | NS | 0.01 |  |
| Treatments vs Controls | | | NS | 0.06 | NS | NS |  | NS | NS | NS | 0.01 |  |

Where: -

F_1_& F_2_: 80% NP (100:50 kg NP ha^‑1^) fertigation in 8 and 10 similar doses respectively;

F_3_& F_4_: 100% NP (125:62.5 kg NP ha^-1^) fertigation in 8 and 10 similar doses respectively;

C_1_(SFM):Surface flood with 100% NP (125:62.5 kg NP ha^-1^) through manual broadcasting in 2 similar doses (Control 1);

C_2_: SDF at 20 cm depth with 100% N (100:50 kg NP ha^‑1^) in 10 similar doses& emitter spacing at 20 cm (Control 2);

SDF: Subsurface drip fertigation;

DAS: days after sowing

Supplementary Table5 Effect of lateral depth, emitter spacing and fertigation scheduling on root length density of wheat during 2022-23

| Lateral  depth (L) | Emitter spacing (S) | Fertigation levels (F) | Root length density (cm cm^-3^) | | | | | | | | | |
| --- | --- | --- | --- | --- | --- | --- | --- | --- | --- | --- | --- | --- |
|  |  |  | 90-100 DAS | | | |  | at maturity | | | | |
|  |  |  | 0- 15 cm | 15-30 cm | 30-45 cm | 45-60 cm |  | 0- 15 cm | 15-30 cm | 30-45 cm | 45-60 cm |  |
| L_1_  (25 cm) | S_1_  (30 cm) | F_1_ | 1.303 | 0.970 | 0.557 | 0.283 |  | 0.857 | 0.737 | 0.477 | 0.227 |  |
|  |  | F_2_ | 1.370 | 1.007 | 0.573 | 0.293 |  | 0.867 | 0.750 | 0.483 | 0.230 |  |
|  |  | F_3_ | 1.447 | 1.040 | 0.590 | 0.307 |  | 0.887 | 0.753 | 0.500 | 0.237 |  |
|  |  | F_4_ | 1.467 | 1.043 | 0.597 | 0310 |  | 0.937 | 0.803 | 0.507 | 0.247 |  |
|  | S_2_  (40 cm) | F_1_ | 1.250 | 0.930 | 0.553 | 0.283 |  | 0.843 | 0.730 | 0.467 | 0.213 |  |
|  |  | F_2_ | 1.323 | 0.970 | 0.563 | 0.287 |  | 0.863 | 0.737 | 0.473 | 0.227 |  |
|  |  | F_3_ | 1.400 | 0.983 | 0.580 | 0.300 |  | 0.870 | 0.750 | 0.473 | 0.230 |  |
|  |  | F_4_ | 1.437 | 1.030 | 0.580 | 0.307 |  | 0.893 | 0.783 | 0.500 | 0.230 |  |
| L_2_  (30 cm) | S_1_  (30 cm) | F_1_ | 1.227 | 0.950 | 0.533 | 0.280 |  | 0.840 | 0.723 | 0.463 | 0.217 |  |
|  |  | F_2_ | 1.273 | 0.960 | 0.560 | 0.287 |  | 0.857 | 0.740 | 0.470 | 0.223 |  |
|  |  | F_3_ | 1.387 | 0.973 | 0.573 | 0.297 |  | 0867 | 0.743 | 0.487 | 0.230 |  |
|  |  | F_4_ | 1.410 | 1.020 | 0.580 | 0.303 |  | 0.893 | 0.787 | 0.497 | 0.233 |  |
|  | S_2_  (40 cm) | F_1_ | 1.220 | 0.927 | 0.550 | 0.280 |  | 0.833 | 0.713 | 0.463 | 0.217 |  |
|  |  | F_2_ | 1.263 | 0.960 | 0.565 | 0.283 |  | 0.857 | 0.740 | 0.470 | 0.217 |  |
|  |  | F_3_ | 1.367 | 0.973 | 0.579 | 0.293 |  | 0.867 | 0.747 | 0.483 | 0.227 |  |
|  |  | F_4_ | 1.383 | 1.010 | 0.584 | 0.297 |  | 0.880 | 0.763 | 0.490 | 0.230 |  |
| L_1_ | | | 1.375 | 0.997 | 0.573 | 0.296 |  | 0.877 | 0.755 | 0.485 | 0.230 |  |
| L_2_ | | | 1.316 | 0.972 | 0.566 | 0.290 |  | 0.862 | 0.746 | 0.478 | 0.224 |  |
| S_1_ | | | 1.360 | 0.995 | 0.575 | 0.295 |  | 0.875 | 0.754 | 0.480 | 0.230 |  |
| S_2_ | | | 1.330 | 0.973 | 0.565 | 0.291 |  | 0.863 | 0.745 | 0.476 | 0.223 |  |
| F_1_ | | | 1.250 | 0.944 | 0.550 | 0.282 |  | 0.843 | 0.726 | 0.468 | 0.219 |  |
| F_2_ | | | 1.307 | 0.974 | 0.565 | 0.288 |  | 0.861 | 0.742 | 0.474 | 0.224 |  |
| F_3_ | | | 1.400 | 0.992 | 0.579 | 0.299 |  | 0.873 | 0.748 | 0.486 | 0.231 |  |
| F_4_ | | | 1.424 | 1.026 | 0.584 | 0.304 |  | 0.901 | 0.784 | 0.499 | 0.235 |  |
| C_1_ | | | 1.192 | 0.886 | 0.522 | 0.278 |  | 0.829 | 0.721 | 0.456 | 0.209 |  |
| C_2_ | | | 1.338 | 0.972 | 0.570 | 0.289 |  | 0.864 | 0.742 | 0.479 | 0.206 |  |
| CD (p=0.05) | | |  |  |  |  |  |  |  |  |  |  |
| Lateral depth (L) | | | 0.05 | NS | NS | NS |  | NS | NS | NS | NS |  |
| Emitter spacing (S) | | | NS | NS | NS | NS |  | NS | NS | NS | NS |  |
| Fertigation levels (F) | | | 0.08 | 0.06 | NS | 0.01 |  | 0.04 | 0.03 | NS | NS |  |
| Interactions | | | NS | NS | NS | NS |  | NS | NS | NS | NS |  |
| C_1_ vs C_2_ | | | 0.08 | 0.06 | 0.04 | NS |  | NS | NS | NS | NS |  |
| Treatments vs Controls | | | 0.09 | 0.07 | NS | NS |  | NS | NS | NS | NS |  |

Where: -

F_1_& F_2_: 80% NP (100:50 kg NP ha^‑1^) fertigation in 8 and 10 similar doses respectively;

F_3_& F_4_: 100% NP (125:62.5 kg NP ha^-1^) fertigation in 8 and 10 similar doses respectively;

C_1_(SFM):Surface flood with 100% NP (125:62.5 kg NP ha^-1^) through manual broadcasting in 2 similar doses (Control 1);

C_2_: SDF at 20 cm depth with 100% N (100:50 kg NP ha^‑1^) in 10 similar doses& emitter spacing at 20 cm (Control 2);

SDF: Subsurface drip fertigation;

DAS: days after sowing

Supplementary Table 6 Effect of lateral depth, emitter spacing and fertigation scheduling on nitrogen and phosphorus uptake by cotton and wheat crops

| Lateral  depth (L) | Emitter spacing (S) | Fertigation levels (F) | N uptake  (kg ha^-1^) | | | |  | P uptake  (kg ha^-1^) | | | | |
| --- | --- | --- | --- | --- | --- | --- | --- | --- | --- | --- | --- | --- |
|  |  |  | Cotton (2021) | Cotton (2022) | Wheat (2021-22) | Wheat (2022-23) |  | Cotton (2021) | Cotton (2022) | Wheat (2021-22) | Wheat (2022-23) |  |
| L_1_  (25 cm) | S_1_  (30 cm) | F_1_ | 203.0 | 152.7 | 85.3 | 86.0 |  | 24.6 | 18.1 | 12.9 | 19.6 |  |
|  |  | F_2_ | 208.7 | 159.7 | 87.0 | 90.3 |  | 25.0 | 18.8 | 13.4 | 20.1 |  |
|  |  | F_3_ | 254.3 | 177.3 | 96.0 | 101.3 |  | 26.1 | 22.7 | 14.3 | 21.1 |  |
|  |  | F_4_ | 273.0 | 195.0 | 108.3 | 115.7 |  | 26.2 | 23.0 | 14.8 | 23.2 |  |
|  | S_2_  (40 cm) | F_1_ | 195.3 | 143.3 | 78.3 | 83.7 |  | 24.6 | 16.6 | 12.6 | 17.7 |  |
|  |  | F_2_ | 202.7 | 154.3 | 83.0 | 91.0 |  | 25.5 | 20.1 | 12.6 | 18.2 |  |
|  |  | F_3_ | 250.0 | 167.7 | 94.0 | 102.7 |  | 26.7 | 22.5 | 12.8 | 20.4 |  |
|  |  | F_4_ | 269.0 | 180.7 | 102.3 | 110.7 |  | 25.9 | 23.1 | 14.0 | 23.6 |  |
| L_2_  (30 cm) | S_1_  (30 cm) | F_1_ | 194.3 | 154.7 | 76.3 | 86.0 |  | 24.9 | 18.1 | 11.7 | 17.0 |  |
|  |  | F_2_ | 199.3 | 155.3 | 85.0 | 90.3 |  | 26.6 | 20.3 | 12.1 | 20.5 |  |
|  |  | F_3_ | 243.7 | 165.0 | 93.3 | 101.3 |  | 27.6 | 22.2 | 13.3 | 21.8 |  |
|  |  | F_4_ | 263.0 | 183.3 | 97.7 | 108.7 |  | 27.6 | 23.5 | 13.9 | 23.2 |  |
|  | S_2_  (40 cm) | F_1_ | 190.3 | 134.0 | 76.7 | 88.0 |  | 23.0 | 17.2 | 10.4 | 16.6 |  |
|  |  | F_2_ | 197.3 | 152.0 | 82.7 | 95.7 |  | 24.1 | 17.5 | 11.4 | 17.9 |  |
|  |  | F_3_ | 242.3 | 159.7 | 91.3 | 99.3 |  | 25.5 | 19.1 | 13.5 | 22.0 |  |
|  |  | F_4_ | 255.7 | 173.3 | 96.3 | 103.3 |  | 27.3 | 22.4 | 13.8 | 22.2 |  |
| L_1_ | | | 232.0 | 166.3 | 91.8 | 97.7 |  | 25.6 | 20.6 | 13.4 | 20.5 |  |
| L_2_ | | | 223.3 | 159.7 | 87.4 | 96.6 |  | 25.8 | 20.0 | 12.5 | 20.1 |  |
| S_1_ | | | 229.9 | 167.9 | 91.1 | 97.5 |  | 26.1 | 20.8 | 13.3 | 20.8 |  |
| S_2_ | | | 225.3 | 158.1 | 88.1 | 96.8 |  | 25.3 | 19.8 | 12.6 | 19.8 |  |
| F_1_ | | | 195.8 | 146.2 | 79.2 | 85.9 |  | 24.3 | 17.5 | 11.9 | 17.7 |  |
| F_2_ | | | 202.0 | 155.3 | 84.4 | 91.8 |  | 25.3 | 19.2 | 12.4 | 19.2 |  |
| F_3_ | | | 247.6 | 167.4 | 93.7 | 101.2 |  | 26.5 | 21.6 | 13.5 | 21.3 |  |
| F_4_ | | | 265.2 | 183.1 | 101.2 | 109.6 |  | 26.7 | 23.0 | 14.1 | 23.1 |  |
| C_1_ | | | 172.0 | 134.3 | 74.0 | 81.9 |  | 23.5 | 15.9 | 10.9 | 13.5 |  |
| C_2_ | | | 211.7 | 160.1 | 90.1 | 92.7 |  | 24.9 | 22.5 | 12.5 | 20.1 |  |
| LSD (p=0.05) | | |  |  |  |  |  |  |  |  |  |  |
| Lateral depth (L) | | | 7.6 | NS | NS | NS |  | NS | NS | NS | NS |  |
| Emitter spacing (S) | | | NS | NS | NS | NS |  | NS | NS | NS | NS |  |
| Fertigation levels (F) | | | 8.7 | 9.9 | 8.3 | 7.1 |  | 1.3 | 1.1 | 1.1 | 1.4 |  |
| Interactions | | | NS | NS | NS | NS |  | NS | NS | NS | NS |  |
| C_1_ vs C_2_ | | | 12.3 | 17.3 | 8.5 | 9.9 |  | 1.3 | 1.7 | NS | 1.6 |  |
| Treatments vs controls | | | 12.6 | 17.7 | 8.7 | 10.1 |  | 1.3 | 1.7 | 1.9 | 1.7 |  |

Where: -

For Cotton: F_1_ & F_2_: 100% N (112.5 kg N ha^-1^) fertigation in 10 and 14 similar doses;

F_3_ & F_4_: 125% N (140 kg N ha^-1^) fertigation in 10 and 14 similar doses respectively;

C_1_: Surface flood with 100% N (105 kg N ha^‑1^) through manual broadcasting in 2 similar doses (Control 1);

C_2_: SDF at 20 cm depth with 100% N (112.5 kg N ha^-1^) in 10 similar doses& emitter spacing at 20 cm (Control 2);

For wheat: F_1_ & F_2_: 80% NP (100:50 kg NP ha^‑1^) fertigation in 8 and 10 similar doses respectively;

F_3_ & F_4_: 100% NP (125:62.5 kg NP ha^-1^) fertigation in 8 and 10 similar doses respectively;

C_1_: Surface flood with 100% NP (125:62.5 kg NP ha^-1^) through manual broadcasting in 2 similar doses (Control 1);

C_2_: SDF at 20 cm depth with 100% N (100:50 kg NP ha^‑1^) in 10 similar doses& emitter spacing at 20 cm (Control 2);

SDF: Subsurface drip fertigation
